# Morphological characterization of trichomes shows enormous variation in shape, density and dimensions across the leaves of 14 *Solanum* species

**DOI:** 10.1093/aobpla/plab071

**Published:** 2021-11-02

**Authors:** Sakshi Watts, Rupesh Kariyat

**Affiliations:** 1 Department of Biology, University of Texas Rio Grande Valley, Edinburg, TX 78539, USA; 2 School of Earth Environmental and Marine Sciences, University of Texas Rio Grande Valley, Edinburg, TX 78539, USA

**Keywords:** electron microscopy, *glandular*, non-glandular, solanum, trichomes

## Abstract

Trichomes are the epidermal appendages commonly observed on plant surfaces including leaves, stem and fruits. Plant trichomes have been well studied as a structural plant defence designed to protect plants against abiotic and biotic stressors such as UV rays, temperature extremities and herbivores. Trichomes are primarily classified into glandular and non-glandular trichomes, based on the presence or absence of a glandular head. The plant genus *Solanum* is the largest genus of family Solanaceae that houses ~3500 species of ecological and economic importance have a diverse set of trichomes that vary in density and morphology. However, due to the incomplete and contradictory classification system, trichomes have subjective names and have been largely limited to be grouped into glandular or non-glandular types. Through this study, we did a complete workup to classify and characterize trichomes on both adaxial and abaxial leaf surface of 14 wild and domesticated species of the genus *Solanum*. Using electron microscopy, statistical analyses and artistic rendition, we examined finer details of trichomes and measured their density and dimensions to compile a detailed data set which can be of use for estimating the variation in trichome types, and their density, with consequences for understanding their functional roles. Our study is the first of its kind that provides us with a better and well-defined classification, density and dimension analysis to complete the morphological classification of trichomes on both leaf surfaces of a diverse range of members in *Solanum* genus.

## Teaser

Our study comprehensively documents the morphological variation of trichomes (plant hairs) on both adaxial and abaxial surface of 14 *Solanum* species (mixture of wild and cultivated species), thus providing a catalogue of major trichome types in *Solanum* genus. For each trichome type, the detailed characterization, nomenclature, density on leaf surface (per mm^2^ of leaf surface), dimensions (in µm) and a line art (visual tool) have been provided, which can act as an updated data source and reference tool for future studies.

## Introduction

Plant surfaces show spectacular variation in the shape, size, location, function and origin of epidermal projections (Werker 2000). The most important and well-studied among these are trichomes: unicellular or multicellular appendages (hair-like structures) originating from epidermal cells of various plant parts including leaves, stems and flowers (Oksanen 2018), and developing outwards (Werker 2000). Trichomes are distributed almost universally in the plant kingdom and exhibit dramatic variation in their morphology (Seithe and Sullivan 1990; van Dam *et al.* 1999; Adedeji *et al.* 2007; Kang *et al.* 2010; Nurit-Silva and Fatima Agra 2011; Munien *et al.* 2015; Mehdi Talebi *et al.* 2018) and density (Mehdi Talebi *et al.* 2018), both intra- (van Dam *et al.* 1999; Kang *et al.* 2010; Munien *et al.* 2015) and interspecifically (Seithe and Sullivan 1990; Navarro and Oualidi 1999; Mannethody and Purayidathkandy 2018; Mehdi Talebi *et al.* 2018; Yu *et al.* 2018), and also among and between related and distant plant families (Kariyat *et al.* 2018; Deore 2020; Watts and Kariyat 2021). For example, Munien *et al.* (2015) found four types of trichomes (glandular, non-glandular dendritic, non-glandular bicellular and non-glandular multicellular) in *Withania somnifera* (intraspecific); Yu *et al.* (2018) found a great variation in trichome morphology, dimensions, distribution and density among seven *Mentha* species (interspecific); Deore (2020) identified variations in trichomes among 20 species belonging to 12 different plant families and most of the species were reported to have trichomes ranging from unicellular to multicellular, conical to elongated, smooth to grooved, thin to thick walled and with or without a flat disc at the base. Further, Tsujii *et al.* (2016) found tremendous variation in trichome leaf dry mass per area in the plant tissue of *Metrosideros polymorpha* at different elevations (Tsujii *et al.* 2016). Clearly, within flowering plants, trichomes are both ubiquitous and morphologically diverse.

Trichomes, in general, are considered as one of the first line of defences possessed by plants to protect against abiotic stresses such as UV rays, water loss, temperature extremities (Ehleringer 1982; Li *et al.* 2018; Oksanen 2018) and herbivore damage (Kaur and Kariyat 2020a, b; Watts and Kariyat 2021). Moreover, the leaf trichomes can also act as mechanoreceptors for detection of insects on leaf surface as observed in case of *Arabidopsis* (Zhou *et al.* 2017). In addition to defence-related functions, trichomes play a role in water usage strategies through maintenance of leaf water content and stomatal traits to name a few. For example, Pan *et al.* (2021) showed higher percentage increase of leaf water content in epiphytic plants with trichomes (Pan *et al.* 2021). Additionally, trichomes can play role in translocation and homeostasis of minerals in plants (Blamey *et al.* 1986, 2015; Li *et al.* 2021). But the relationships of such traits with trichome types and measurements such as density and dimensions are poorly understood.

Broadly, trichomes can be classified into glandular (presence of glandular head) and non-glandular (absence of glandular head) (Werker 2000). Both glandular (Tang *et al.* 2020) and non-glandular (Karabourniotis *et al.* 2019) trichomes have been well documented to protect plants either by production of chemicals in their glandular heads or by their sturdy structure that assist plants to adapt and/or protect from environmental conditions such as UV radiations and cold stresses. Glandular trichomes deter herbivory by physically entrapping herbivore into sticky exudates (Tingey and Laubengayer 1981; Neal *et al.* 1990; Elle *et al.* 1999; Zalucki *et al.* 2002), secreting defensive chemical compounds such as proteinase inhibitors (Peiffer *et al.* 2009), production of volatile organic compounds (Avé *et al.* 1987; Murungi *et al.* 2016) or by altering herbivore body odour after providing a sugar-rich first meal (Weinhold and Baldwin 2011). On the other hand, non-glandular trichomes in the *Solanum* species are mostly spike-like structures which deter herbivory primarily by deterring herbivore movement, feeding and oviposition (Corsi and Bottega 1999; Kennedy 2003; Løe *et al.* 2007; Dalin *et al.* 2008; Sletvold *et al.* 2010; Tian *et al.* 2012; Weigend *et al.* 2018; Kariyat *et al.* 2019). Additionally, non-glandular trichomes can cause post-feeding damage to caterpillars by rupturing of caterpillars’ peritrophic membrane (gut lining; Kariyat *et al.* 2017; Andama *et al.* 2020). Moreover, some plant species also possess stinging hairs (trichomes with stinging cells which contain irritant fluids) which act as hypodermal syringes and can cause various allergic reactions such as pain, itching, oedema and visible dermal reactions to mammalian herbivores (Ensikat *et al.* 2021).

Glandular trichomes are more pliable, so may not cause physical damage (Kariyat 2017, 2019) but can be toxic and can release chemicals to intoxicate herbivores (Hare 2005) and attract predators of herbivores in association with carcasses of herbivores (LoPresti *et al.* 2015). Contrary to this, non-glandular trichomes are usually devoid of toxins but their sharp and edgy structure can cause physical damage to herbivores. Thus, while the size and structure of the apical gland can inform about the amount of toxins and their content, knowledge about various types (e.g. unbranched vs. stellate) and sizes of non-glandular trichomes can appraise us about their functional significance—either in deterring herbivory or protecting against abiotic stressors. More specifically, in case of non-glandular trichomes, we now have multiple lines of evidence on how variation in trichome type and density differentially defend them against insect herbivores (Cho *et al.* 2017; Kariyat *et al.* 2017, 2019; Watts and Kariyat 2021). For instance, Watts and Kariyat (2021) found significant variation in trichome density on abaxial and adaxial leaf surface of 11 Solanaceae species and demonstrated that this variation has functional consequences for caterpillar growth and feeding.

Clearly, a detailed examination and characterization of trichome morphology (even on abaxial and adaxial surfaces) can have multiple benefits, including a reliable and non-contradictory nomenclature of trichomes, understanding the prominent trichome types found in nature and their diversity—which later can be explored for defensive functions against different herbivores, and abiotic stressors, with possible implications for our efforts to produce better defended plants for sustainable agriculture (Andama *et al.* 2020).

Solanaceae is one of the most important plant families consisting of 90 genera and ~3000–4000 ecologically and economically important species which are found in all habitats ranging from dry deserts to wet tropical rainforest and have growth habits ranging from small ephemeral herbs to large perennial trees (Knapp, 2004). Among all the genera in Solanaceae, the genus *Solanum* contributes ~75 % of all species (Symon 1981; Knapp 2002). *Solanum* genus exhibits tremendous variation and diversity of trichomes; for instance, widely studied and domesticated crops such as tomato (*Solanum lycoperiscum*) and tobacco (*Nicotiana tabacum*) have different types of glandular trichomes, while wild weeds such as silverleaf nightshade (*Solanum elaeagnifolium*) and Carolina horsenettle (*Solanum carolinense*) possess only non-glandular trichomes (Peiffer *et al.* 2009; Weinhold and Baldwin 2011; Burrows *et al.* 2013; Kariyat *et al.* 2018). A general convention in trichome literature is to reduce the diversity of trichome types by constraining them to just types of glandular and non-glandular, while these types are quite diverse and are often complicated to resolve. Moreover, this basic classification also fails to explain the huge variation among subtypes of glandular and non-glandular trichomes, and consequently potential to explore their function.

Luckwill (1943) was the first to classify trichomes of *Lycopersicon* into seven distinct types (four glandular subtypes and three non-glandular subtypes) of trichomes based on their length, number of stalk and base cells, and the presence or absence of gland. Following Luckwill (1943), Uphof (1962) in a summary for different methods of classification of trichomes concluded that the final classification is still subjective. After that, Payne (1978) provided us with a glossary to name various trichomes or structures found in trichomes to improve trichome nomenclature. Later, Channarayappa *et al.* (1992) revised the trichome classification by Luckwill (1943) and the revised classification is used frequently in trichome-related studies. While these studies have served as a model for trichome morphology assessment, we used scanning electron microscopy, and artistic rendering along with the previous classical classification systems (Roe 1971) and the glossary provided by Payne (1978) to characterize and classify trichomes on both adaxial (upperside) and abaxial (lowerside) leaf surface of 14 representative species from *Solanum*.

## Materials and Methods

### Plant materials

A mixture of wild and domesticated species of genus *Solanum* (14 species in total) were included in the study. We bought seeds of forest bitterberry (*Solanum anguivi*; Product code: Y5SSSOIN), porcupine tomato (*S. pyracanthos*; Product code: Y5SSSOPY), African eggplant (*S. macrocarpon*; Product code: Y5SSSOMC), bittersweet nightshade (*S. dulcamara*; Product code: Y5SSS0DU), lance-leaved nightshade (*S. lanceifolium*; Product code: Y5SSSOLA), potato tree (*S. grandiflorum*; Product code: Y5SSSOGR), tzimbalo (*S. caripense*; Product code: Y5SSSOCA), devil’s fig (*S. asperolanatum*; Product code: Y5SSSOAS), *S. taeniotrichum* (Product code: Y5SSSOTA) from rarepalmseeds.com; tomato (*S. lycopersicum*; Variety: Valley Girl F1) seeds from Johnnyseeds.com; garden huckleberry (*S. melanocerasum*; Brand: Palm Beach Medicinal Herbs), easter eggplant (*S. ovigerum*; Brand: Helens Garden) and Turkish orange eggplant (*S. aethiopicum*; Seller: Seedville USA) from amazon.com, and Aubergine (*S. melongena*; Shikou hybrid Eggplant; Item: 52568-PK-P1) from parkseed.com.

Seeds of all the species were sown in potting mixture (Sunshine professional growing mix: Sun Gro Horticulture Canada Ltd, Agawam, MA, USA; Tayal *et al.* 2020) filled trays (12.5″ × 7.5″ × 2″) and kept in controlled environmental conditions (26 °C temperature, ~50 % relative humidity and 16:8 light dark cycle). Germinated seedlings were transplanted in plastic pots (5″ × 4″ × 4″) with similar soil media and environmental conditions and were watered regularly. For electron microscopy, plants of 4–6 weeks of age post-transplanting with at least 10–12 fully developed leaves were used. Young and fully expanded leaves from randomly selected individuals (for each species, sample size varied by treatment, details below) were used for microscopy experiments.

### Desktop scanning electron microscope

To capture images from both abaxial and adaxial of leaves for trichome morphology (*n* = 3–11 plants per side per species), dimension measurements (*n* = 3–11 plants per side per species) and density analysis (*n* = 3–11 plants per side per species), a desktop scanning electron microscope (DSEM; SNE- 4500 Plus Tabletop; Nanoimages LLC, Pleasanton, CA, USA; Watts *et al.* 2021) was employed. Circular leaf discs (0.63 cm in diameter) of fresh leaf samples (collection method detailed above) were excised from the plants using a hole punch. No chemical treatments (e.g. glutaraldehyde; Kariyat *et al.* 2017), critical drying or sputter coating, were done to the leaf samples, and fresh leaf discs mounted on the aluminium stubs using double-sided carbon tape were directly inserted in the DSEM for scanning and image processing. For more details on operational procedures and methodology of DSEM, see Watts and Kariyat (2021) and Watts *et al.* (2021).

### Trichome morphology assessment

Fresh leaf samples (*n* = 3–11 plants per side per species) as described above were used and magnified ranging from 45× to 1000× depending on trichome type and size, to achieve maximum resolution to extract finer details of trichomes. Images of different trichome types from both abaxial and adaxial surface of leaves were captured at different angles in 3D and later used to classify them. Payne (1978; consisting of glossary for different shapes and structures of/in trichomes), Roe (1971; consisting of terminology for commonly found *Solanum* trichomes) and Werker (2000; glandular trichome characterization based on structure of secretory head) were the major literature used to characterize trichomes post-image acquisition.

### Trichome density assessment

To determine the trichome density from both leaf surfaces of all the species (*n* = 3–11 plants per side per species), sample preparation was done as described above. The images for trichome count were consistently captured at 60× magnification which contains ~5.32 mm^2^ leaf area measured using ‘Nanoeye’ software linked to DSEM. We calculated the trichome density 1 mm^2^ as follows (Chavana *et al.* 2021; Watts and Kariyat 2021):


$${\text{Trichome density (1 mm}}^{\text{2}}\text{)}=\text{Number of trichomes in the image taken at 6}0\times \text{magnification}/\text{5}.\text{32}$$

### Trichome dimension measurements

While scanning the leaf samples (*n* = 3–11 plants per side per species), once the image achieved maximum resolution visually, scanning was paused using ‘Nanoeye’ software associated with DSEM and dimensions of various trichome types were measured by tracing trichomes by straight line in ‘M. tools’ in ‘Nanoeye’ software. For non-glandular trichomes, length of spikes from base to tip was measured, and in case of glandular trichomes, length of trichome from base to tip and diameter of bulb containing glandular secretions were measured using the measurement tool embedded in the software (Fig. 7). Magnification was altered among samples depending on trichome type at the best resolution.

**Figure 1.**
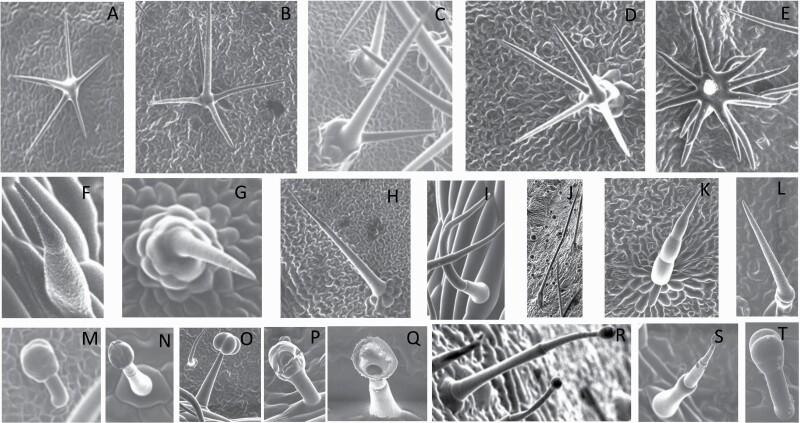
The three major trichome types found in the study includes (A–E) stellate non-glandular trichomes, (F–L) simple non-glandular trichomes and (T–U) glandular trichomes. Figure shows scanning electron microscopic images of (A) porrect-stellate multiradiate non-glandular hair with subulate rays (2–7 in number) and with short central ray on adaxial leaf surface of *Solanum lanceifolium*, (B) porrect-stellate multiradiate non-glandular hair with subulate rays (3–7 in number) and with long central ray on adaxial leaf surface of *S. ovigerum*, (C) bifurcated basilatus non-glandular hair with subulate rays (one shorter than the other) on adaxial leaf surface of *S. anguivi*, (D) multitangulate multiradiate stellate non-glandular hair with subulate rays (2–6 in number) with the presence of pedestal on adaxial leaf surface of *S. aethiopicum*, (E) porrect-geminate stellate multiradiate non-glandular hair with subulate rays (2–16 in number) and short central ray on abaxial leaf surface of *S. melongena*, (F) osteolate non-glandular hair with multicellular stalk on abaxial leaf surface of *S. dulcamara*, (G) subulate basilatus non-glandular trichome with distinct subsidiary cells on adaxial leaf surface of *S. aethiopicum*, (H) subulate basilatus non-glandular hair with multicellular jointed stalk and multicellular base on adaxial leaf surface of *S. caripense*, (I) hooked subulate non-glandular hair on abaxial leaf surface of *S. lycopersicum*, (J) subulate non-glandular hair with multiseriate base and tall pedestal on *S. grandiflorum*, (K) subulate non-glandular hair with multicellular jointed stalk, multicellular base and distinct subsidiary cells on adaxial leaf surface of *S. melanocerasum*, (L) subulate non-glandular hair with pulvinate base and a pedestal on adaxial leaf surface of *S. macrocarpon*, (M) glandular hair with single stalk and neck cell and large quadricellular globular head on adaxial leaf surface of *S. anguivi*, (N) glandular hair with single stalk and neck cell and large doliform globular head on adaxial leaf surface of *S. lanceifolium*, (O) glandular hair with large quadricellular globular head and single stalk cell on abaxial leaf surface of *S. lycopersicum*, (P) glandular hair with large globular head and single stalk cell on abaxial leaf surface of *S. macrocarpon*, (Q) glandular hair with large globular head and single stalk cell on abaxial leaf surface of *S. grandiflorum*, (R) subulate basilatus glandular hair with multicellular jointed stalk and small glandular tip on abaxial leaf surface of *S. taeniotrichum*, (S) subulate glandular hair with multicellular jointed stalk and small glandular tip on adaxial leaf surface of *S. taeniotrichum*, (T) glandular hair with small globular head on adaxial leaf surface of *S. ovigerum.*

**Figure 2.**
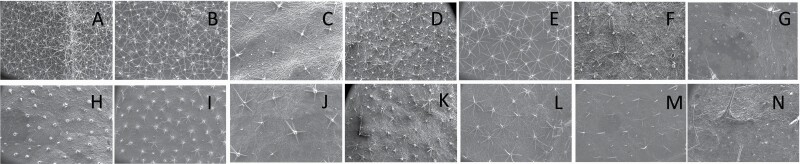
Scanning electron microscopic (SEM) images captured at 60× magnification of abaxial (A–G) and adaxial (H–N) leaf surface of (A, H) *Solanum aethiopicum*, (B, I) *Solanum anguivi*, (C, J) *Solanum lanceifolium*, (D, K) *Solanum melongena*, (E, L) *Solanum pyracanthos*, (F, M) *Solanum ovigerum* and (G, N) *Solanum grandiflorum* with the presence of stellate non-glandular trichomes as one of the major trichome types.

**Figure 3.**
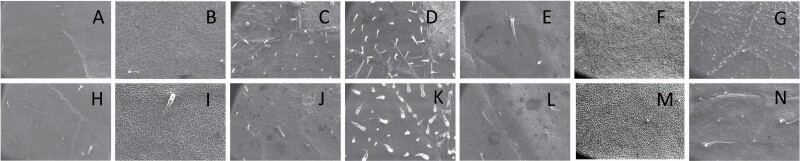
Scanning electron microscopic (SEM) images captured at 60× magnification of abaxial (A–G) and adaxial (H–N) leaf surface of (A, H) *Solanum macrocarpon*, (B, I) *Solanum melanocerasum*, (C, J) *Solanum asperalanatum*, (D, K) *Solanum taeniotrichum*, (E, L) *Solanum caripense*, (F, M) *Solanum dulcamara* and (G, N) *Solanum lycopersicum*, with stellate non-glandular trichome absent as a major trichome type.

**Figure 4.**
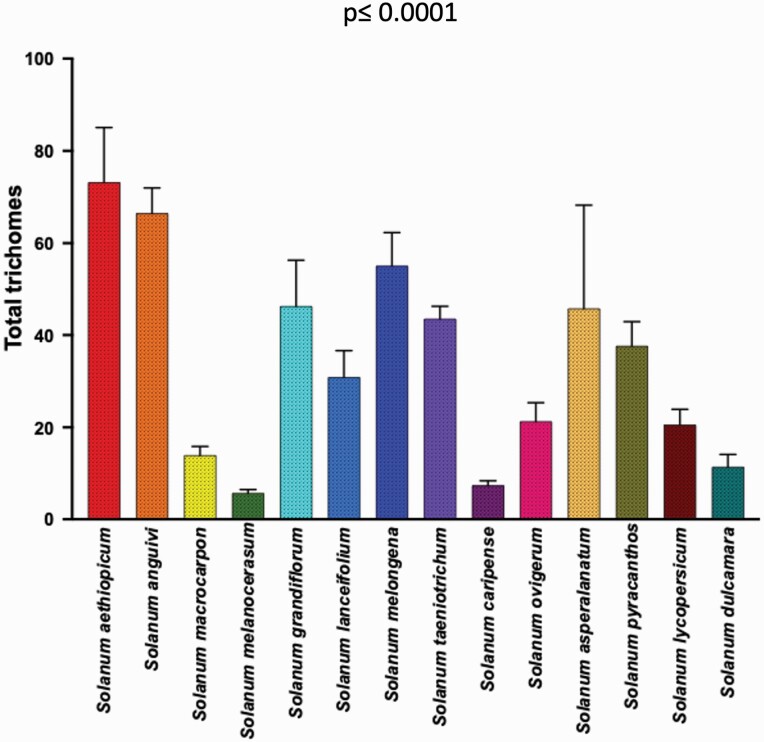
Significant variation in total trichome density (generalized regression; *P* = <0.0001) among 14 *Solanum* species.

**Figure 5.**
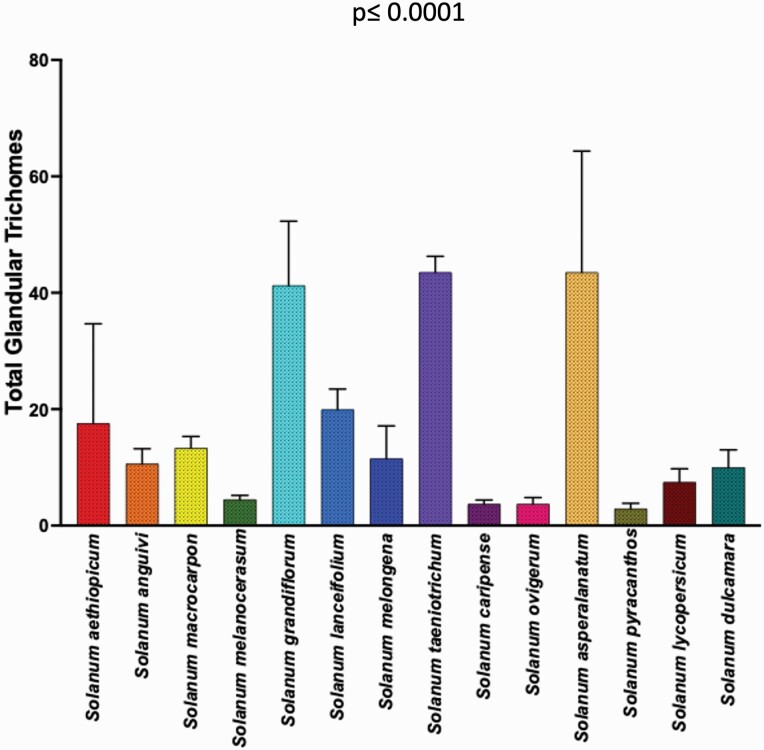
Significant variation in total glandular trichome density (generalized regression; *P* = <0.0001) among 14 *Solanum* species.

**Figure 6.**
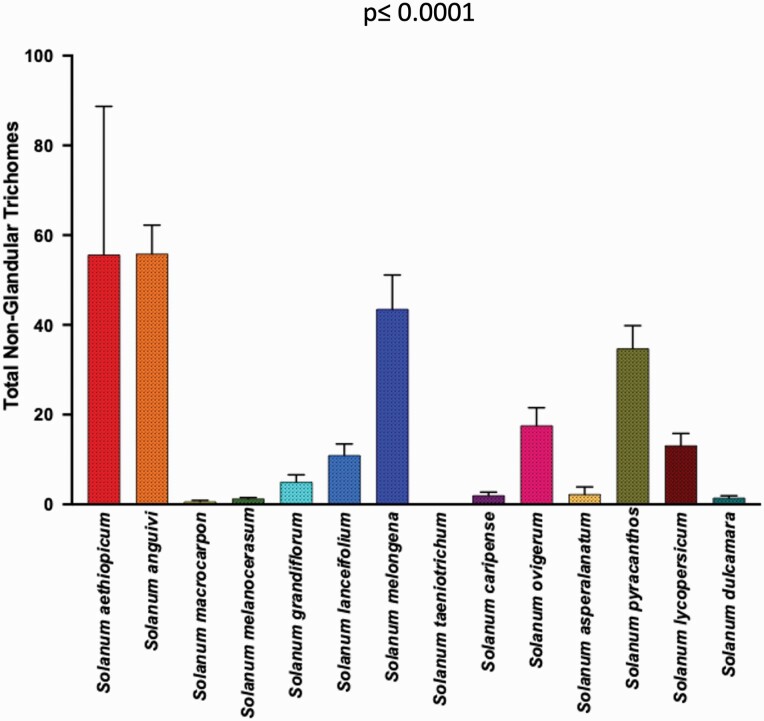
Significant variation in total non-glandular trichome density (generalized regression; *P* = <0.0001), among 14 *Solanum* species.

**Figure 7.**
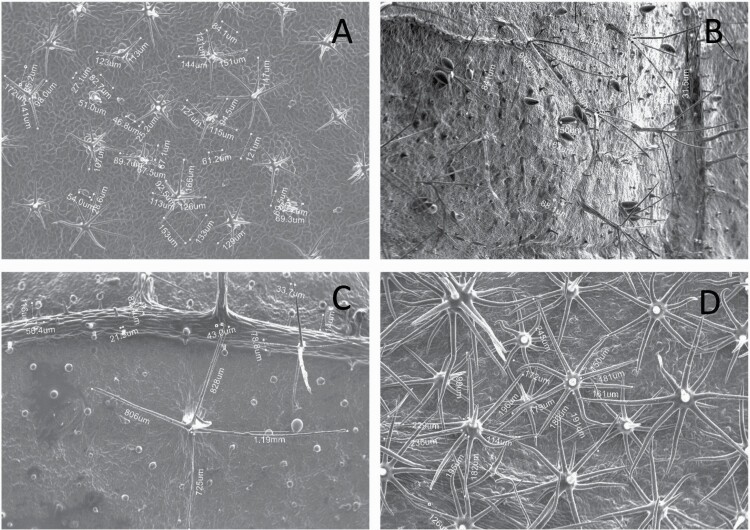
Dimension measurement of different trichome types on (A) adaxial leaf surface of *Solanum anguivi* at 100×, (B) adaxial leaf surface of *S. aperolanatum* at 45×, (C) abaxial leaf surface of *S. melongena* at 150×, (D) abaxial leaf surface of *S. grandiflorum* at 60×, by tracing the trichome projections (length of hair and diameter of bulb in case of glandular trichomes, and length of hair/spike in case of non-glandular trichomes) using the ‘Nanoeye’ software.

#### Line art

The SEM images were used to draw the trichomes manually on paper. The paper was scanned to make a digital copy and the trichomes were traced using a size 4 solid circle brush (Wacom Intuos Pro Digital Graphic Drawing Tablet; Adobe Photoshop). Further, custom brushes in Adobe Photoshop were used to create non-uniform surface of few trichome types. The images were saved into transparent PNG files and inserted into tables with the base of trichomes is on the bottom of the image.

### Analysis

Our goal was to characterize and document the trichome types in these 14 species, and to define the finer details on individual trichome types, and their dimensions. Using previous publications as a composite reference, we classified the trichome types using images that reflected its most detailed morphological features. The nomenclature of trichomes was decided by following a checklist of features in the order described below: major shape of the trichome; glandular/non-glandular trichome type (italicized); additional minute morphological specifications. Further, additional features have also been added as a separate column to know more details of each trichome type. For morphological representation of trichome types, line art was created for each individual trichome type, by a graphical artist.

Following this, mean trichome density for each trichome type or trichome types as broad groups (glandular; simple non-glandular; stellate non-glandular) was calculated manually from images at 60× magnification. 60× magnification includes 5.32 mm^2^ of leaf area, and thus to get density of trichomes in 1 mm^2^ of area, the trichomes density at 60× magnification was divide by a factor of *5.32*. The mean ± SE of trichome density has been incorporated in Tables 2 and 3. And, to test whether species and trichomes varied across the 14 species of interest, we also ran a generalized regression analysis with Poisson distribution with species and types (total, glandular and non-glandular) and their interaction as factors, and trichome number as the response variables. Tukey’s *post hoc* tests were conducted to examine pairwise comparisons. All analyses were carried out using JMP15 (SAS Inc., Cary, NC, USA) software and the plots were built using GraphPad Prism (La Jolla, CA, USA). And finally mean ± SE total length and mean ± SE of the diameter of glands on glandular trichomes, and mean ± SE spike length in case of non-glandular trichomes were measured.

**Table 1.** List of terminology used to define trichomes in the study.

Terminology	Definition (compiled from previously published literature)
Attenuate	Long and gradually tapering
Base	Lowermost part of the trichome
Basilatus	Emerging from a broad base
Bifurcated	Divided into two branches
Brevicolate	Short-necked
Compound	Having multiple rays
Cruciate	Shaped as a cross with four equal arms
Doliform	Barrel-shaped
Falcate	Sickle-shaped
Glandular	Has secretory/excretory function
Hooked	Bent/incurved apex-shaped
Head	Has an enlarged terminal portion
Jointed	Presence of apparent articulation
Mamilla	Nipple-shaped projection
Multiradiate	Multi-rayed
Multitangulate	Rays at many angles
Muticous	No pointed tip/ blunt tip
Neck	Middle cell of a uniseriate glandular hair
Non-glandular	Without secretory/excretory function
Osteolate	Thighbone-shaped with many cells having swollen ends
Ovoid	Egg-shaped with attachment at larger end
Pedestal	Raised base to which hairs are attached
Porrect-geminate	Similar to porrect-stellate but consisting of two whorls of rays one over the other
Porrect-stellate	Resemblance with porrect rays of cacti with multiple horizontal rays and a central ray
Pulvinate	Swollen base
Pustulated	Blistered surface
Quadricellular	Four cells
Sessile	Without stalks (for glands)
Setiform	Bristle-shaped
Simple	Unbranched
Smooth	Without any surface irregularities
Stalk	Supporting part of a hair
Stellate	Star-shaped
Subsidiary cells	Neighbouring base cells
Subulate	Awl-shaped
Tufted/penicillate	Branched from a base
Uniseriate	Single rows/columns of cells
Verrucate	Warty-shaped

**Table 2.** Detailed morphological characterization (major shape and additional features) of trichomes on adaxial leaf surface along with pictorial representation, density and dimensions of trichomes in 14 *Solanum* species in the study (‘very rare’ in density table indicates the absence of certain trichome types while counting trichomes at 60×; however, the trichome was present very rarely in very few images which were not at 60× magnification; blank values in density and dimensions table indicate that the density of that particular trichome types has already been included in the density of a broader trichome type, such as density of all glandular trichomes, or density of simple non-glandular trichome types or density of all stellate trichome types as one number rather than individual density of all subtypes of these trichomes; ‘no data’ in dimension table indicates the lack of dimensions of that trichome; asterisks in dimension table indicate the multiplication sign showing the length of trichome multiplied by width of gland of trichome, in case of glandular trichomes).

Species	Serial number	Trichome types (major shape)	Additional features (simple smooth uniseriate—S; compound smooth uniseriate—C; simple pustulated uniseriate—P)	Line art of morphology	Density (average ± standard error; trichome number per mm^2^ leaf area)	Dimensions (in µm; length in case of non-glandular trichome; length * width of gland in case of glandular trichome; average ± standard error)
Ethiopian eggplant (*Solanum aethiopicum*)	1.	subulate basilatus *non-glandular* trichome with distinct subsidiary cells	S	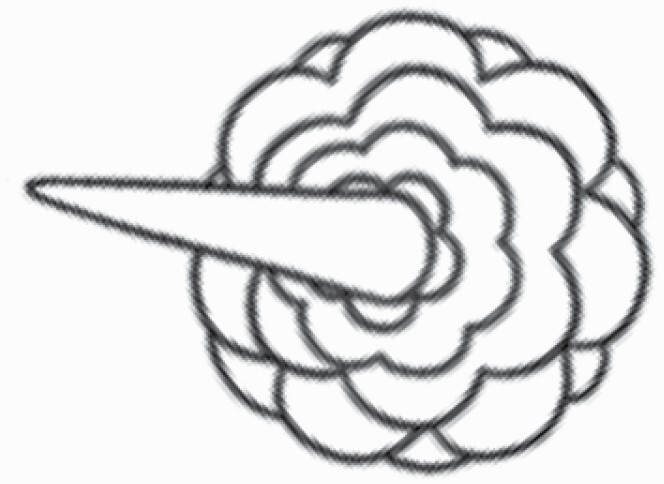	4.73 ± 1.02	153.71 ± 15.67
	2.	multitangulate multiradiate stellate *non-glandular* hair with subulate rays (2–6 in number) with the presence of pedestal	C	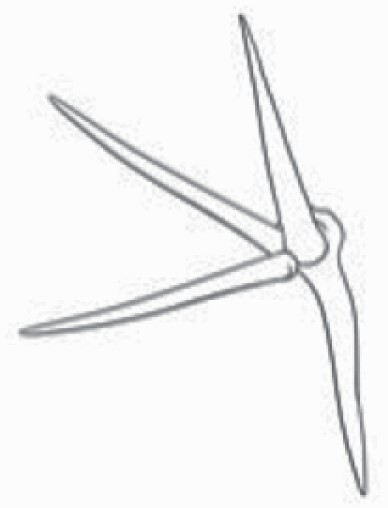	1.06 ± 0.73 (#2 + #3)	185.08 ± 7.29 (#2 and #3)
	3.	bifurcated *non-glandular* hair with subulate rays with the presence of pedestal	C	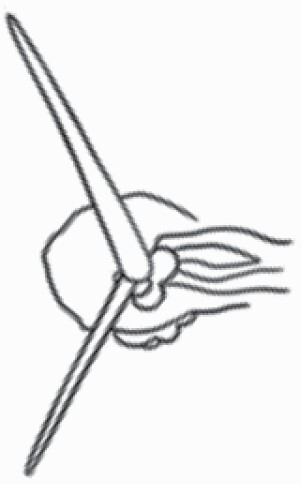	see #2	see #2
	4.	*glandular* hair with small globular head on the top	S	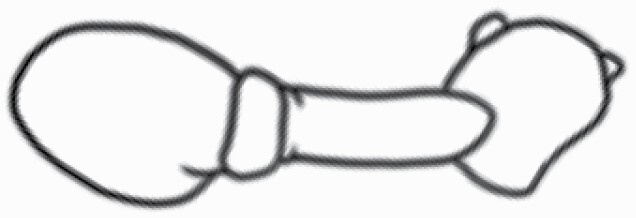	4.88 ± 1.40	65.25 ± 5.58 * 25.08 ± 1.88
Forest bitterberry (*Solanum anguivi*)	5.	*glandular* hair with single stalk and neck cell and large quadricellular globular head	S	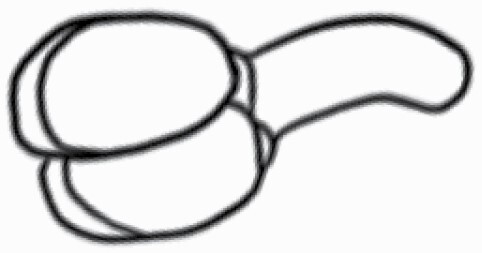	3.50 ± 0.60	59.58 ± 3.13 * 27.11 ± 1.69
	6.	subulate basilatus *non-glandular* hair	S	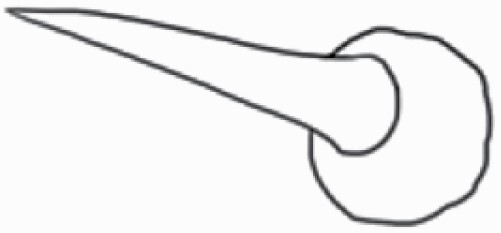	2.40 ± 0.90	93.14 ± 9
	7.	bifurcated basilatus *non-glandular* hair with subulate rays (one shorter than the other)	C	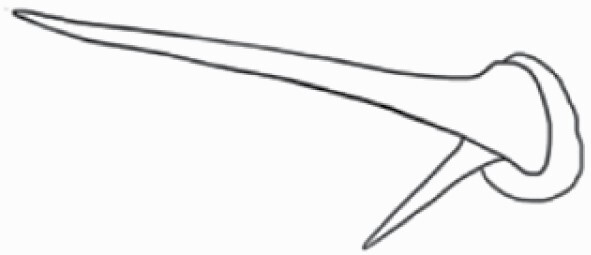	see #8	see #8
	8.	porrect-stellate multiradiate *non-glandular* hair with subulate rays (3–10 in number) with long central ray and pedestal	C	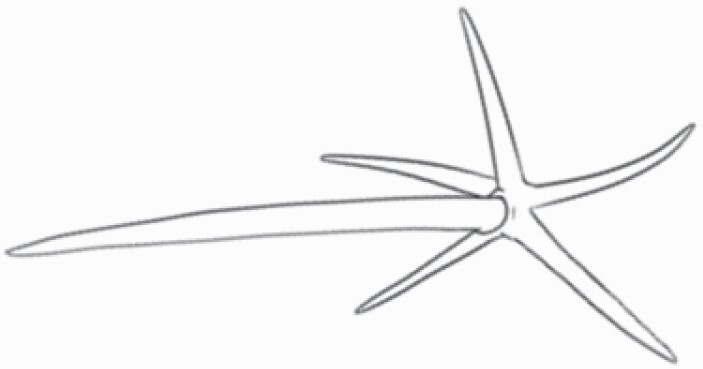	5.92 ± 0.96 (#7 + #8)	133.01 ± 5.39 (#7 and #8)
Lance-leaved nightshade (*Solanum lanceifolium*)	9.	porrect-stellate multiradiate *non-glandular* hair with subulate rays (2–7 in number) and short central ray	C	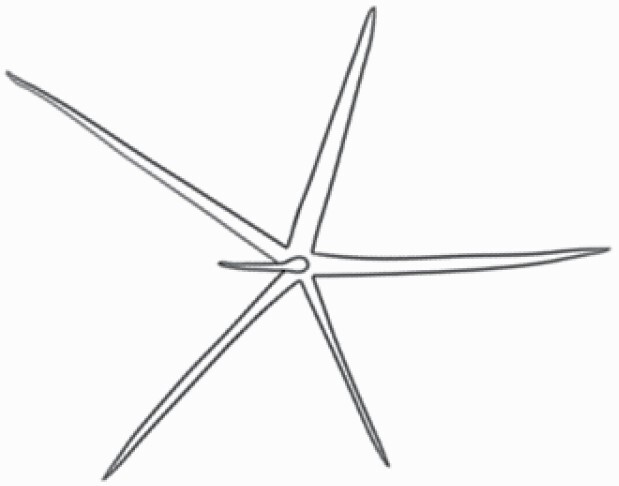	0.72 ± 0.14 (#9 + #10)	193.75 ± 10.22 (#9 and #10)
	10.	porrect-stellate multiradiate *non-glandular* hair with subulate rays (2–7 in number) and long central ray	C	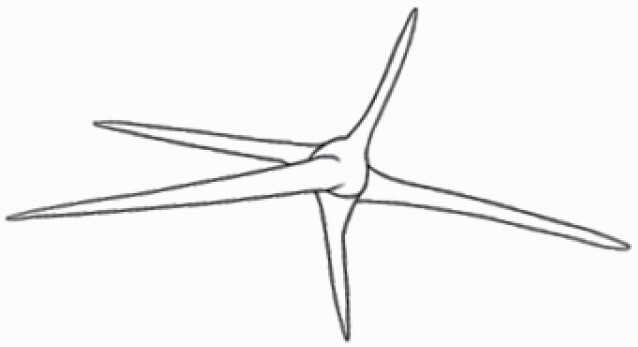	see #9	see #9
	11.	*glandular hair* with single stalk and neck cell and large doliform globular head	S	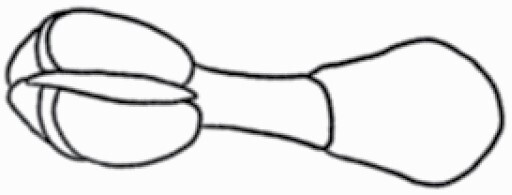	see #12	see #12
	12.	*glandular* hair with large globular head	S	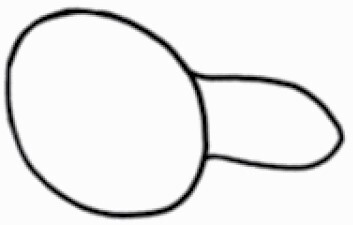	1.45 ± 0.21 (#11 + #12)	66.46 ± 7.19 * 28.64 ± 2.67 (#11 and #12)
Garden tomato (*Solanum lycopersicum*)	13.	attenuate *glandular* hair with small glandular tip	S	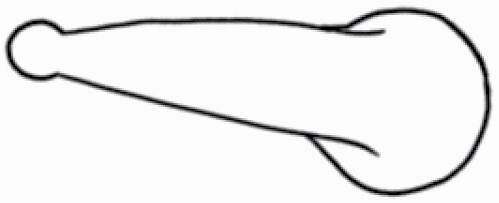	0.55 ± 0.15	63.55 ± 6.35 * 9.85 ± 1.25
	14	*glandular* hair with large globular head	S	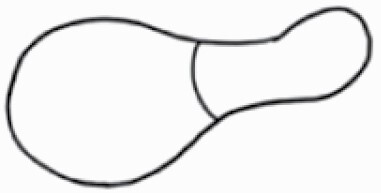	0.44 ± 0.11	58.74 ± 3.18 * 25.38 ± 2.23
	15.	acuminate *glandular* hair with bicellular stalk and small glandular tip	S	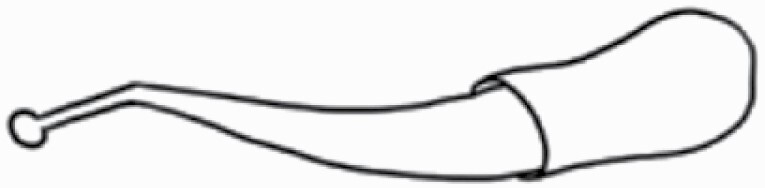	0.05 ± 0.05	327 ± 93.30
	16.	hooked *non-glandular* hair	S	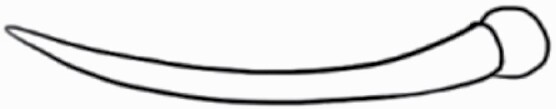	0.97 ± 0.22	218.21 ± 18.24
	17.	attenuate *non-glandular* hair with multicellular jointed stalk and multicellular base	S	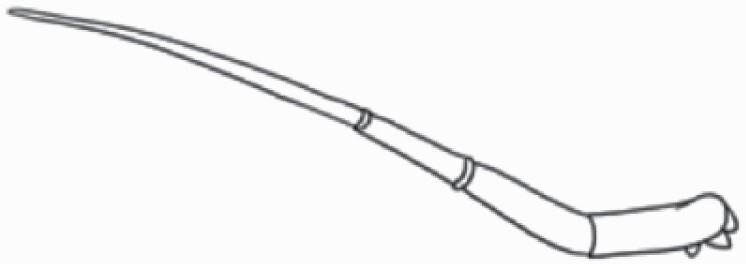	0.17 ± 0.05	327.33 ± 93.30
	18.	attenuate *non-glandular* hair with jointed multicellular stalk	S	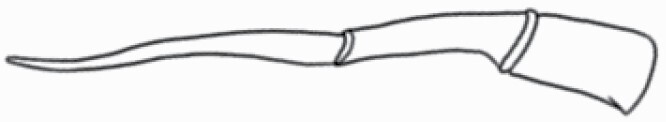	0.12 ± 0.06	436.33 ± 44.63
African eggplant (*Solanum macrocarpon*)	19.	attenuate basilatus *glandular* hair with small glandular tip	S	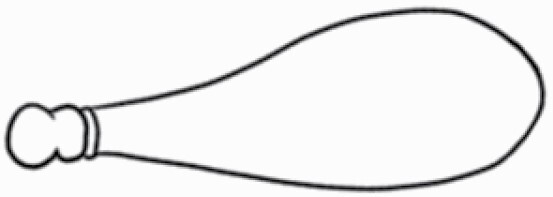	very rare	no data
	20.	subulate *non-glandular* hair with pulvinate base and a pedestal	P	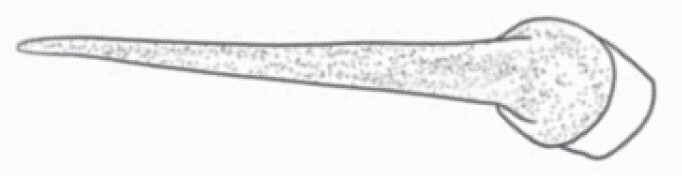	0.22 ± 0.09	288
	21.	*glandular* hair with multicellular large globular head and single stalk cell	S	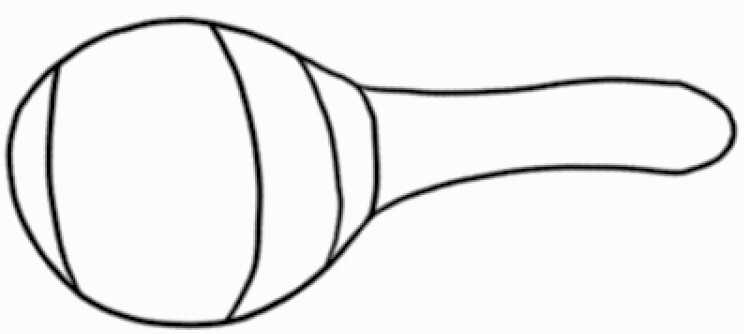	1.88 ± 0.36	72.05 ± 6.99 * 24.23 ± 1.55
Huckleberry (*Solanum melanocerasum*)	22.	subulate *non-glandular* hair with multicellular jointed stalk, multicellular base and distinct subsidiary cells	S	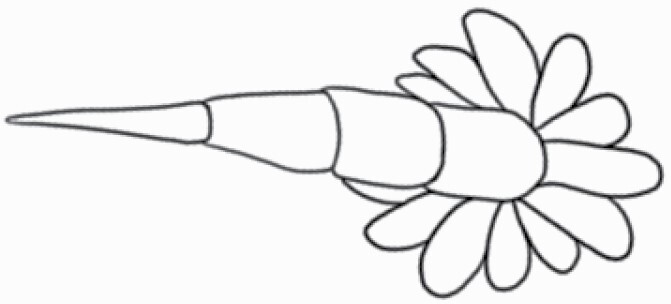	0.23 ± 0.07	421.42 ± 43.01
	23.	subulate *glandular* hair with multicellular jointed stalk, multicellular base, distinct subsidiary cells and small glandular tip	S	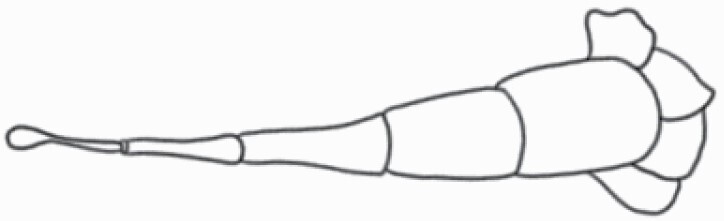	0.02 ± 0.02	461.8 ± 26.03 * 20.68 ± 3.67
	24.	*glandular* hair with large globular head, single stalk cell and no neck cell	S	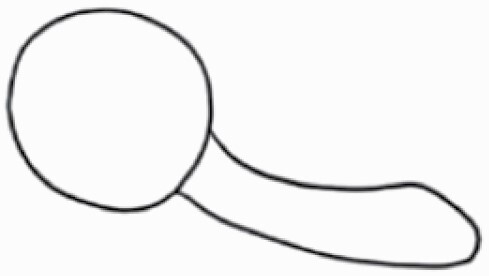	0.77 ± 0.18	78.81 ± 3.11 * 32.37 ± 1.49
Potato tree (*Solanum grandiflorum*)	25.	subulate *non-glandular* hair with multiseriate base and tall pedestal	S	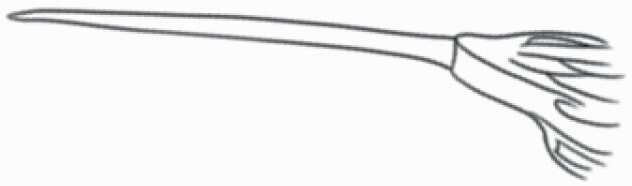	0.47 ± 0.47	583.83 ± 121.39
	26.	*glandular* hair with large globular head and single stalk cell	S	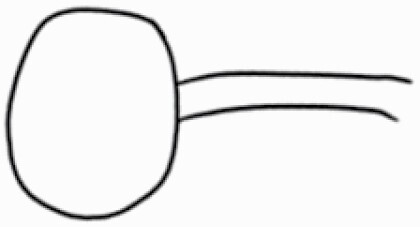	14.57 ± 6.48	80.55 ± 12.72 * 32.21 ± 4.34
	27.	ovoid sessile *non-glandular* hair	S	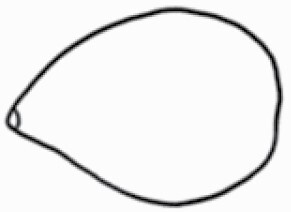	0.19 ± 0.19	no data
	28.	attenuate basilatus *glandular* hair with small glandular tip	S	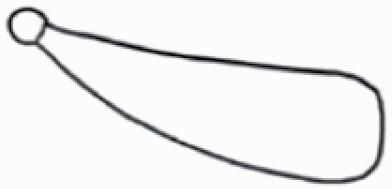	0.19 ± 0.19	256 * 25.2
	29.	setiform *non-glandular* hair with multicellular base and a pedestal	S	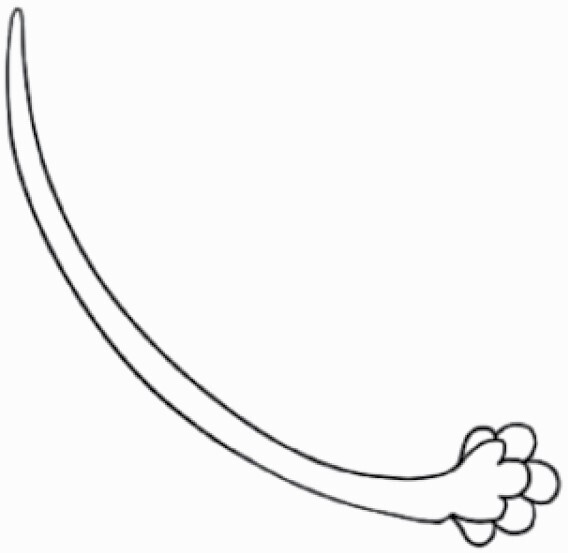	0.94 ± 0.75	549.25 ± 96.85
Eggplant (*Solanum melongena*)	30.	porrect-geminate stellate multiradiate *non-glandular* hair with subulate rays (2–16 in number) and short central ray	C	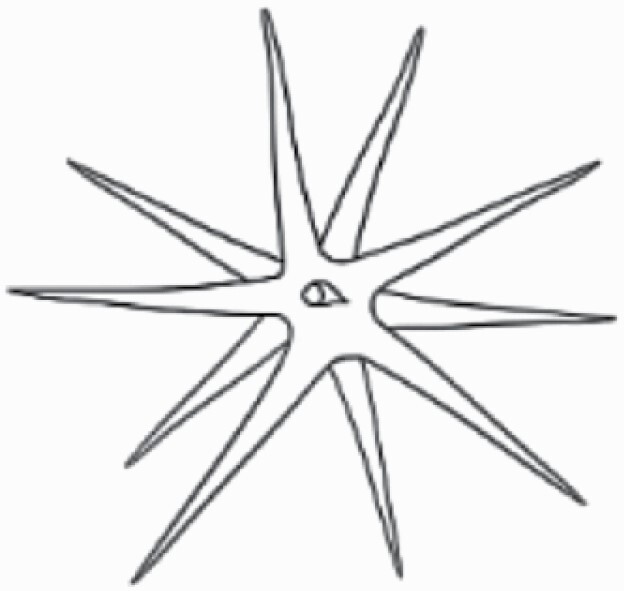	8.34 ± 1.26	168 ± 7.46
	31.	subulate *non-glandular* hair with pulvinate base and pedestal	S	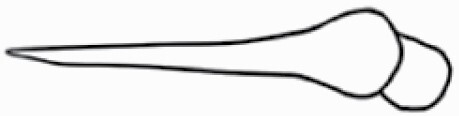	0.19 ± 0.19	64.4 ± 14
	32.	*glandular* hair with large globular head	S	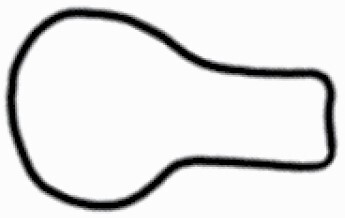	0.41 ± 0.20	47.3 ± 0.9 * 13.1 ± 0.85
*Solanum taeniotrichum*	33.	subulate *glandular* hair with multicellular jointed stalk and small glandular tip	S	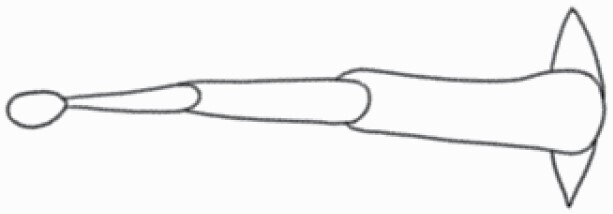	7.20 ± 0.76	385 ± 75 * 48.1 ± 23.8
	34.	*glandular* hair with small globular head	S	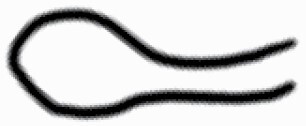	0.44 ± 0.35	no data
Pepino lloron (*Solanum caripense*)	35.	*glandular* hair with small globular head	S	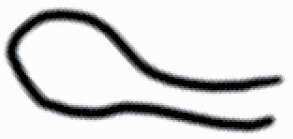	0.75 ± 0.11	52.3 ± 14.2 * 32.2 ± 12.5
	36.	subulate basilatus *non-glandular* hair with multicellular jointed stalk and multicellular base	S	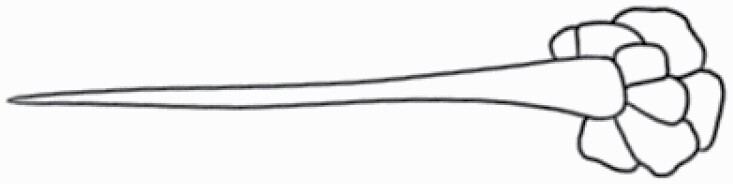	0.88 ± 0.19	431.44 ± 73.48
	37.	subulate *non-glandular* hair with multicellular jointed stalk	S	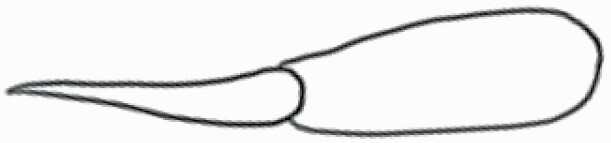	0.12 ± 0.06	287 ± 107
Easter white eggplant (*Solanum ovigerum*)	38.	*glandular* hair with small globular head	S	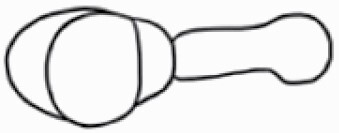	0.44 ± 0.22	54.3 ± 7.17 * 22.4 ± 4.14
	39.	porrect-stellate multiradiate *non-glandular* hair with subulate rays (3–7 in number) and with long central ray	C	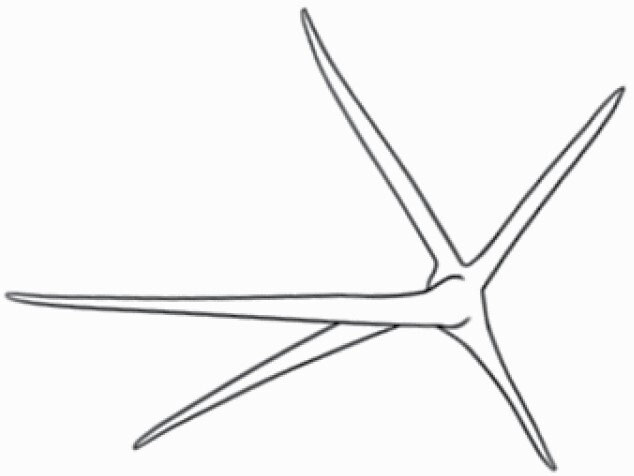	1.38 ± 0.56	198 ± 10.8
	40.	subulate *non-glandular* hair with pulvinate base	S	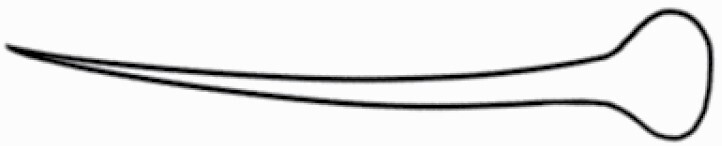	2.13 ± 0.49	153.84 ± 42.38
*Solanum asperolanatum*	41.	*glandular* hair with large globular head, single stalk cell and no neck cell	S	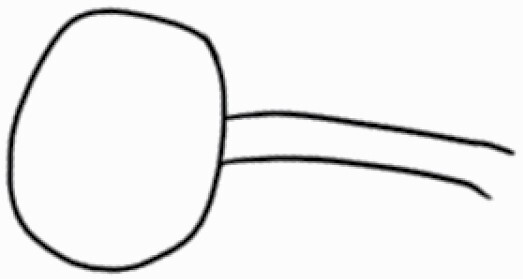	17.30	68.9 ± 5.4 * 20.6 ± 10.9
	42.	ovoid sessile *non-glandular* hair	S	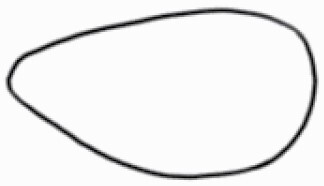	2.25	105 ± 55
	43.	tufted/penicillate *non-glandular* hair with multiseriate long pedestal	C	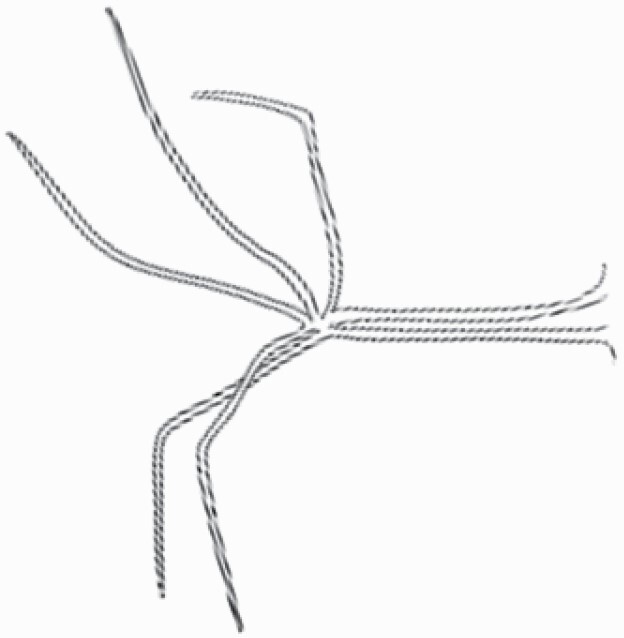	1.31	918 ± 30.7
Porcupine tomato (*Solanum pyracanthos*)	44.	*glandular* hair with small globular head	S	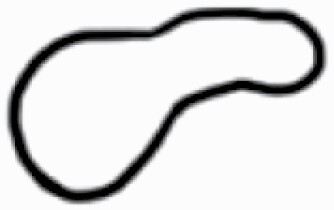	0.62 ± 0.24	48.6 ± 2.87 * 28 ± 2.12
	45.	porrect-stellate multiradiate *non-glandular* hair with subulate rays (2–12 in number) and short central ray	C	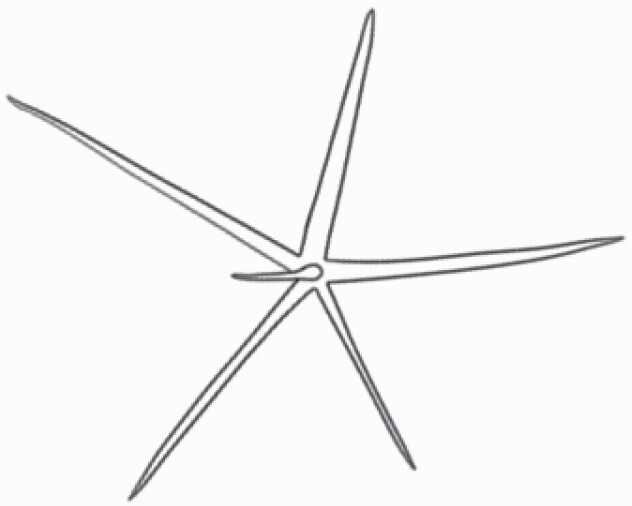	4.13 ± 1.02	199 ± 6.61
	46.	subulate *non-glandular* hair with multicellular stalk and base cells	S	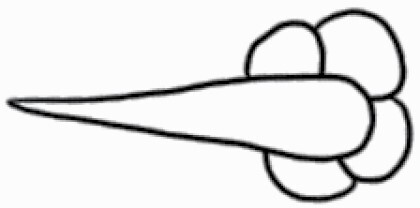	0.60 ± 0.20	107 ± 15.6
Bittersweet nightshade (*Solanum dulcamara*)	47.	subulate basilatus *non-glandular* hair with multicellular stalk	S	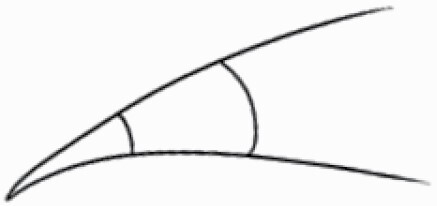	0.52 ± 0.10	158 ± 14
	48.	*glandular* hair with small globular head	S	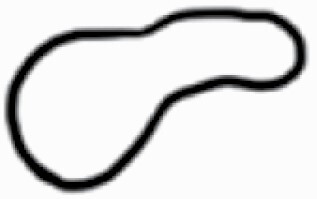	0.19 ± 0.11	no data

**Table 3.** Detailed morphological characterization (major shape and additional features) of trichomes on adaxial leaf surface along with pictorial representation, density and dimensions of trichomes in 14 *Solanum* species in the study (‘very rare’ in density table indicates the absence of certain trichome types while counting trichomes at 60×; however, the trichome was present very rarely in very few images which were not at 60× magnification; blank values in density and dimensions table indicate that the density of that particular trichome types has already been included in the density of a broader trichome type, such as density of all glandular trichomes, or density of simple non-glandular trichome types or density of all stellate trichome types as one number rather than individual density of all subtypes of these trichomes; ‘no data’ in dimension table indicates the lack of dimensions of that trichome; asterisks in dimension table indicate the multiplication sign showing the length of trichome multiplied by width of gland of trichome, in case of glandular trichomes).

Species	Serial number	Trichome types	Additional features (simple smooth uniseriate—S; compound smooth uniseriate—C; simple pustulated uniseriate—P)	Line art of morphology	Density (average ± standard error; trichome number per mm^2^ leaf area)	Dimensions (in µm; length in case of non-glandular trichome; length * width of gland in case of glandular trichome; average ± standard error)
Ethiopian eggplant (*Solanum aethiopicum*)	1.	attenuate *glandular* hair with small glandular tip	S	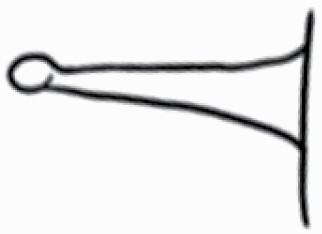	0.86 ± 0.86	101.24 ± 20.42 * 13.66 ± 1.74
	2.	*glandular* hair with large globular head on the top	S	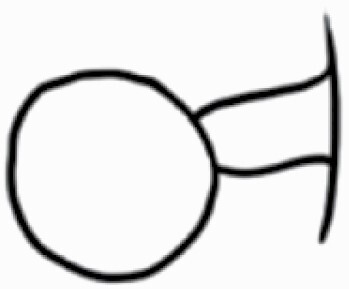	0.52 ± 0.20	52.06 ± 3.83 * 17.11 ± 0.82
	3.	subulate hooked *non-glandular* hair with pulvinate base	S	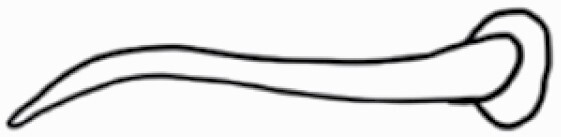	0.82 ± 0.79	88.55 ± 10.79
	4.	porrect-stellate multiradiate *non-glandular* hair with subulate rays (5–10 in number) and short central ray	C	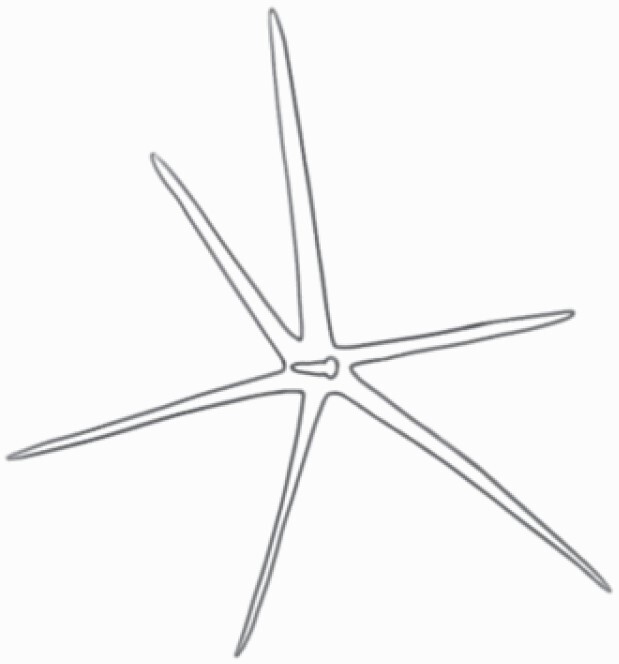	15.26 ± 4.60	241.82 ± 6.36
Forest bitterberry (*Solanum anguivi*)	5.	*glandular* hair with single stalk and neck cell and quadricellular globular head	S	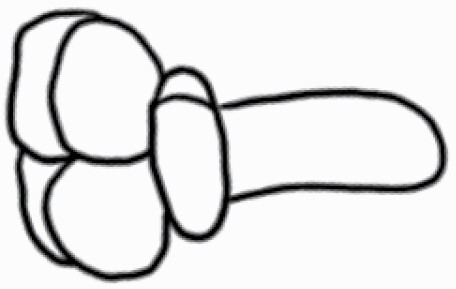	0.72 ± 0.21	46.6 ± 5.1 * 23.35 ± 0.25
	6.	subulate *non-glandular* hair with pulvinate base	S	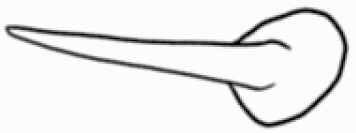	0.19 ± 0.14	113 ± 28.63
	7.	porrect-stellate multiradiate *non-glandular* hair with subulate rays (5–10 in number) and short central ray and pedestal	C	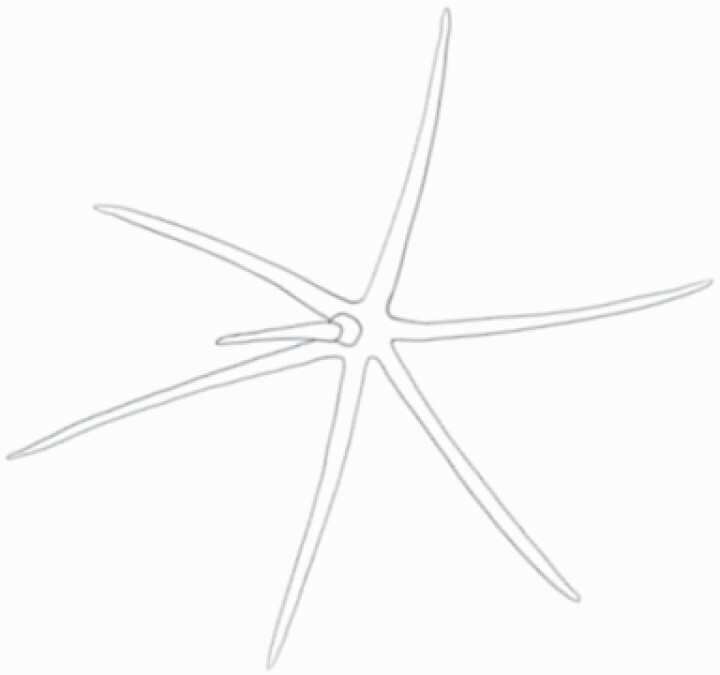	13.00 ± 1.56 (#7 + #8 + #9)	290.85 ± 9.83 (#7, #8 and #9)
	8.	porrect-stellate multiradiate *non-glandular* hair with subulate rays (5–10 in number) with long central ray and pedestal	C	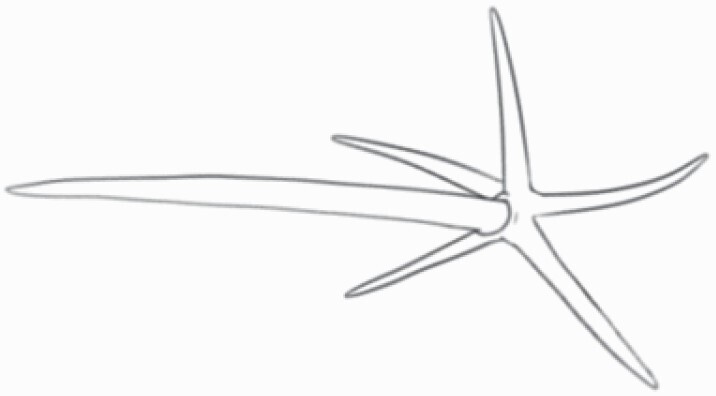	see #7	see #7
	9.	bifurcated basilatus *non-glandular* hair with subulate rays (one shorter than the other)	C	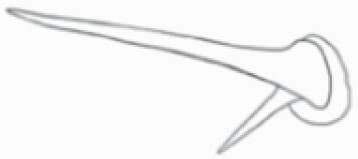	see #7	see #7
Lance-leaved nightshade (*Solanum lanceifolium*)	10.	attenuate *glandular* hair with small globular tip	S	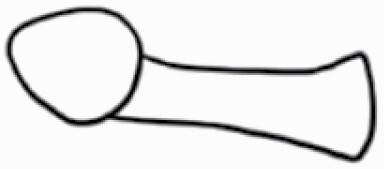	see #12	no data
	11.	verrucate *non-glandular* hair	S	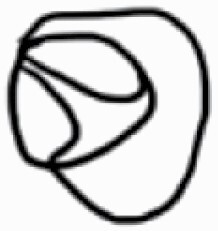	see #12	no data
	12.	*glandular* hair with large globular head	S	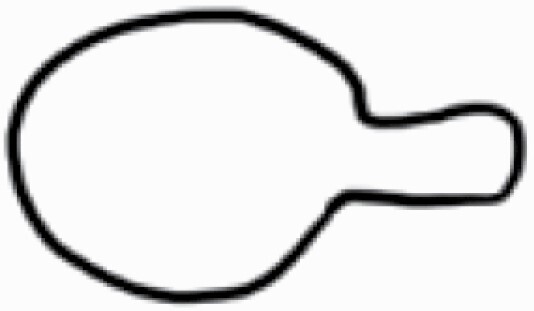	6.04 ± 0.35 (#10, #11 and #12)	40.88 ± 1.22 * 23.79 ± 1.23
	13.	porrect-stellate multiradiate *non-glandular* hair with subulate rays (2–6 in number) and with short central ray	C	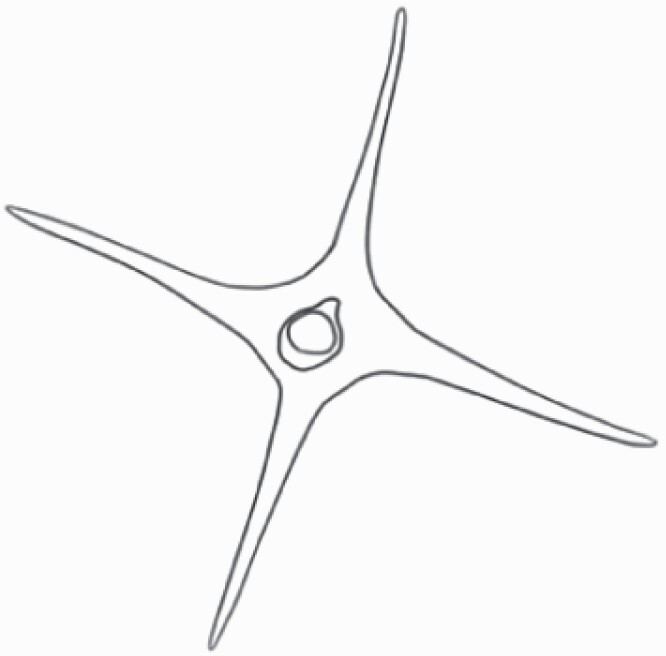	3.38 ± 0.63 (#13+ #14)	156.28 ± 11.76 (#13 and #14)
	14.	bifurcated basilatus *non-glandular* hair with subulate rays (one arm reduced)	S	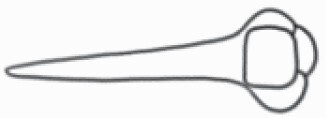	see #13	see #13
Garden tomato (*Solanum lycopersicum*)	15.	*glandular* hair with large quadricellular globular head and single stalk cell	S	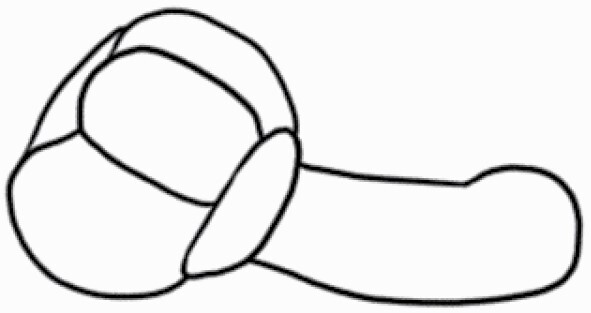	0.94 ± 0.19 (#15 + #16)	42.25 ± 4.69 * 23.32 * 3.07
	16.	*glandular* hair with large quadricellular globular head and single stalk cell	S	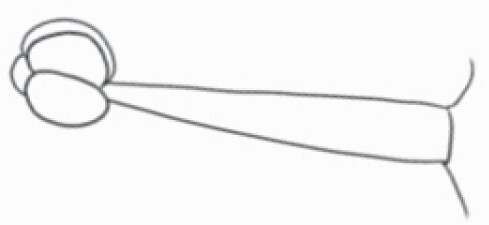	see #15	158 ± 21.38 * 66.57 ± 3.45
	17.	hooked subulate *glandular* hair with multicellular jointed stalk and small glandular tip	S	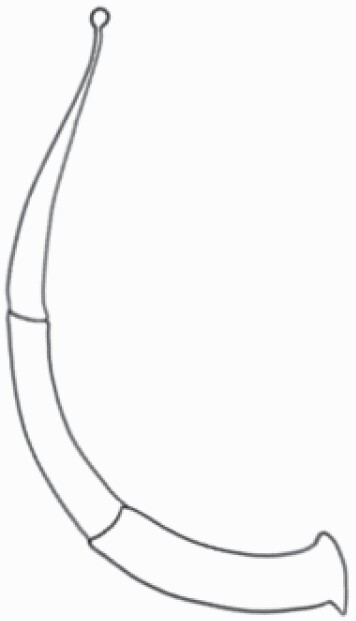	0.30 ± 0.22	378.5 ± 29.23 * 3.50
	18.	hooked subulate *non-glandular* hair	S	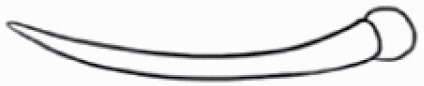	5.4 ± 1.03	204.34 ± 16.40
	19.	attenuate *non-glandular* hair with jointed multicellular stalk	S	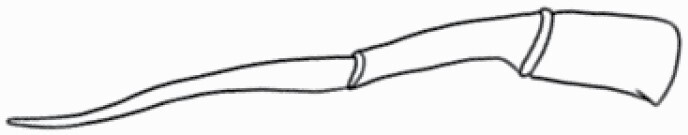	0.35 ± 0.10	884.38 ± 84.30
	20.	attenuate basilatus *glandular* hair with small glandular tip	S	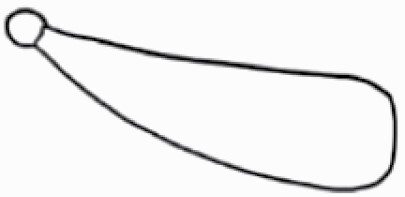	very rare	57.45 ± 8.14 * 7.55 ± 0.91
	21.	mamilla *non-glandular* hair	S	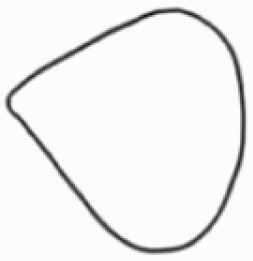	very rare	no data
Gboma (*Solanum macrocarpon*)	22.	*glandular* hair with large globular head and single stalk cell	S	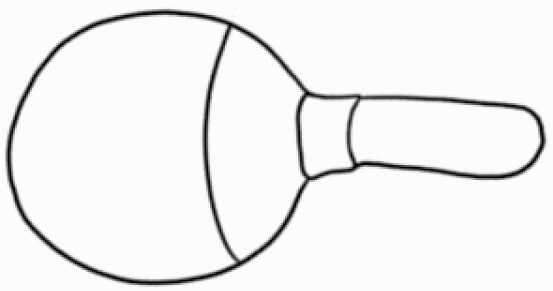	3.03 ± 0.58	72.83 ± 6.79 * 31.69 ± 1.38
	23.	subulate *non-glandular* hair with pulvinate base and a pedestal	P	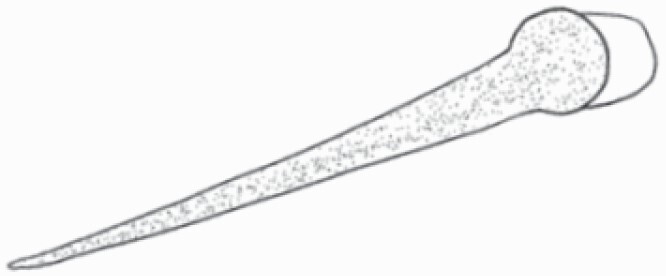	0.02 ± 0.02	301.66 ± 21.6
Huckleberry (*Solanum melanocerasum)*	24.	crescent *non-glandular* hair with multicellular jointed stalk	S	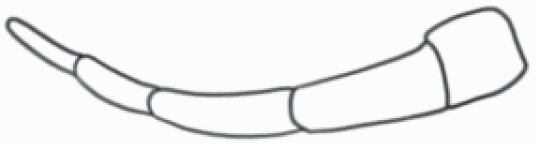	0.18 ± 0.05	312 ± 38.83
	25.	subulate *glandular* hair with multicellular jointed stalk and small glandular tip	S	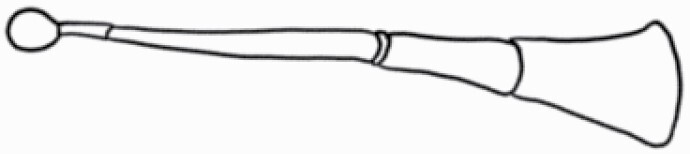	very rare	337.5 ± 39.63 * 24.85 ± 2.7
	26.	*glandular* hair with large globular head, single stalk cell and no neck cell	S	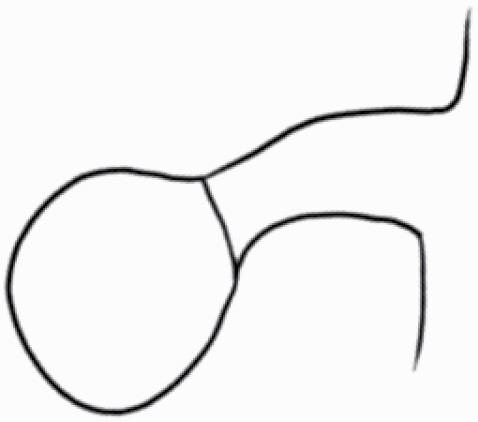	0.81 ± 0.12	73.46 ± 4.52 * 35.09 ± 2.16
Potato tree (*Solanum grandiflorum*)	27.	subulate *non-glandular* hair with multiseriate base and tall pedestal	S	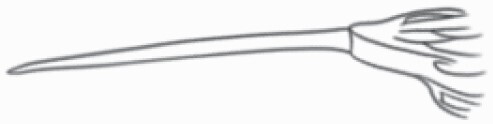	very rare	1194 ± 395.5
	28.	*glandular* hair with large globular head and single stalk cell	S	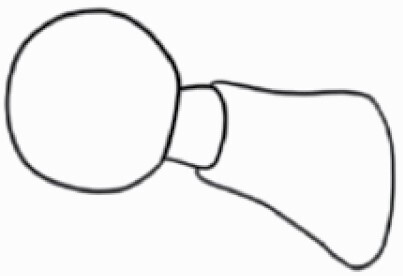	10.15 ± 2.25 (#28 + #29 + #31)	283.2 ± 180.46 * 37.9 ± 7.26 (#28, #29 and #31)
	29.	ovoid sessile *non-glandular* hair	S	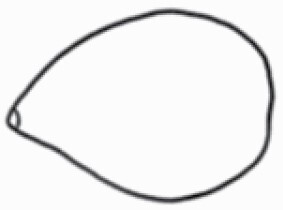	see #28	see #28
	30.	porrect-stellate multiradiate cruciate *non-glandular* hair with subulate rays (4 in number) and short central ray	C	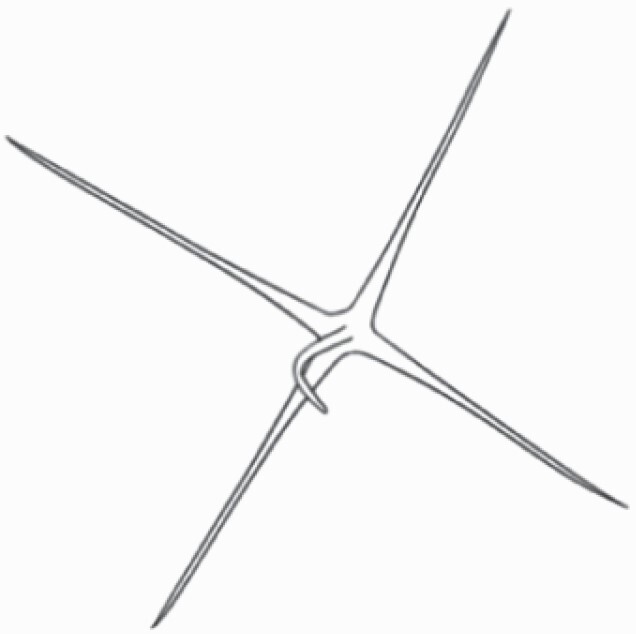	0.47 ± 0.09	887.25 ± 103.31
	31.	*glandular* hair with large globular head, single stalk cell and no neck cell	S	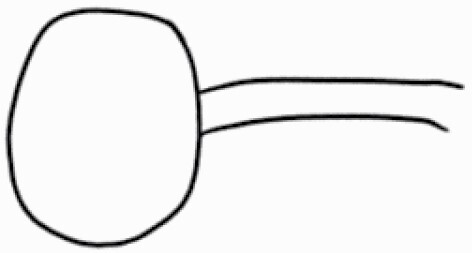	see #28	see #28
	32.	falcate *non-glandular* hair with pulvinate base	S	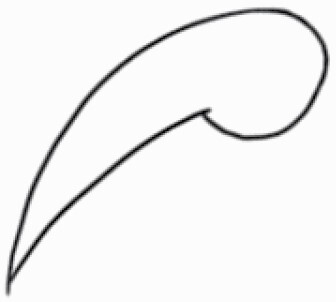	0.56 ± 0.19	no data
*Eggplant* (*Solanum melongena*)	33.	hooked subulate *non-glandular* hair with a pulvinate base	S	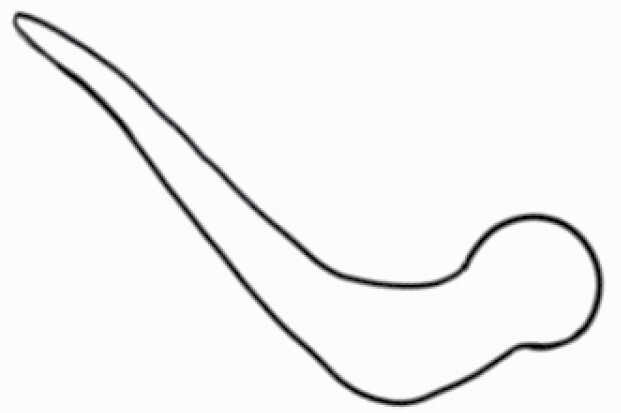	0.08 ± 0.04	103
	34.	porrect-geminate stellate multiradiate *non-glandular* hair with subulate rays (2–16 in number) and short central ray	C	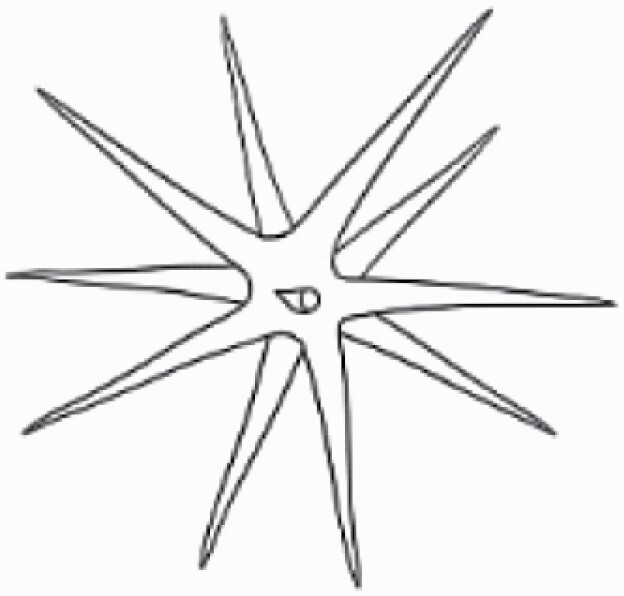	10.12 ± 1.96	211 ± 8.9
	35.	*glandular* hair with large globular head	S	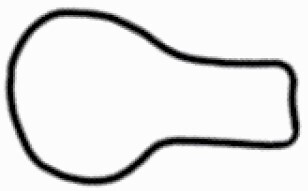	1.48 ± 0.33	44.3 ± 3.86 * 16.9 ± 0.94
*Solanum taeniotrichum*	36.	subulate basilatus *glandular* hair with multicellular jointed stalk and small glandular tip	S	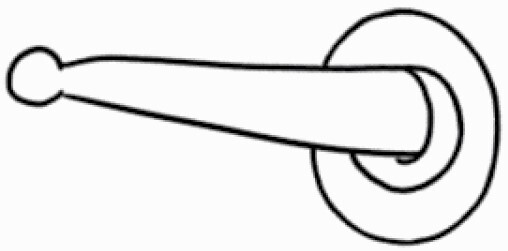	8.21 ± 0.76 (#36+ #37)	344 ± 62 * 21.8 ± 2.33 (#36 and #37)
	37.	subulate basilatus *glandular* hair with multicellular jointed stalk, multicellular base and small glandular tip	S	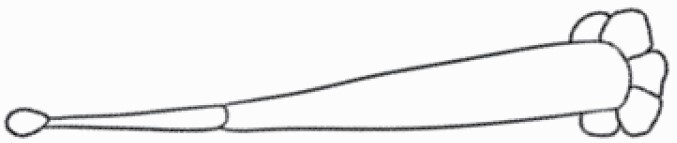	see #36	see #36
	38.	*glandular* hair with small globular head	S	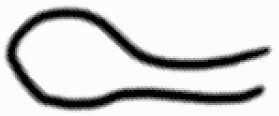	0.44 ± 0.27	51.9 ± 2.55 * 24.4 ± 8.83
	39.	subulate *non-glandular* hair	S	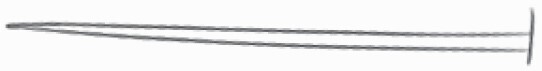	0.06 ± 0.06	1010
Pepino lloron (*Solanum caripense*)	40.	hooked subulate *non-glandular* hair	S	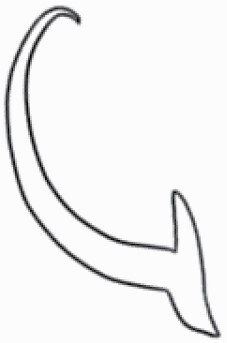	very rare	no data
	41.	subulate *non-glandular* hair	S	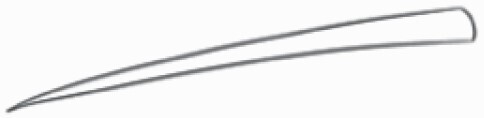	0.66 ± 0.11	318 ± 58.5
	42.	*glandular* hair with large quadricellular globular head	S	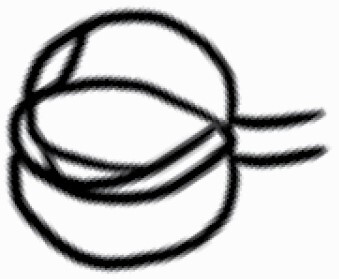	0.66 ± 0.22	53 ± 8 * 22.9 ± 4.65
Easter white eggplant (*Solanum ovigerum*)	43.	subulate *non-glandular* hair with multicellular jointed stalk and multicellular base	S	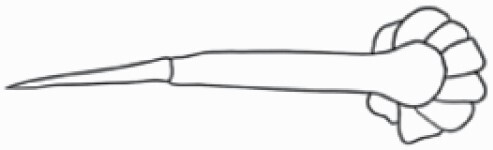	0.23 ± 0.12	63.7 ± 6.35
	44.	falcate *non-glandular* hair	S	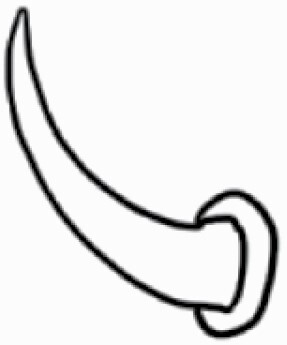	very rare	no data
	45.	porrect-stellate multiradiate *non-glandular* hair with subulate rays (2–12 in number) and with short central ray and pedestal	C	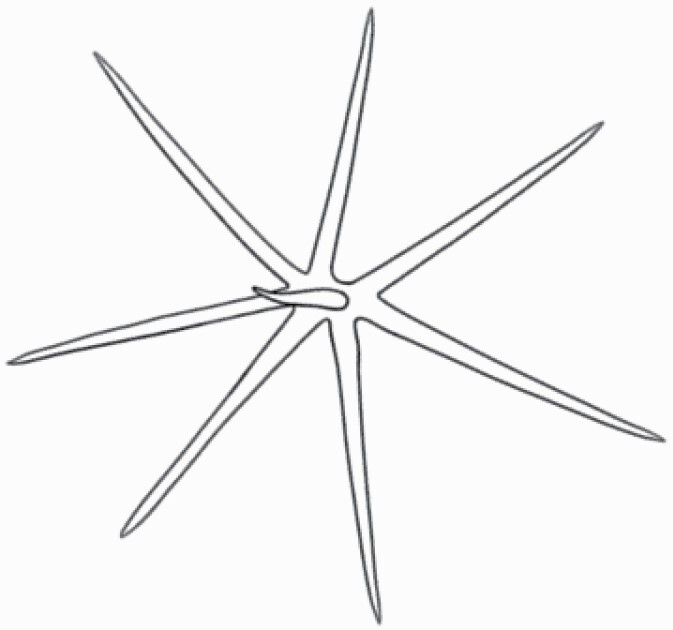	2.4 ± 0.76	229 ± 11.7
	46.	*glandular* hair with small globular head	S	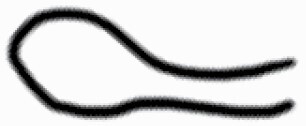	0.90 ± 0.32	49.6 ± 5.32 * 32.7 ± 28
*Solanum asperolantum*	47.	ovoid sessile *non-glandular* hair	S	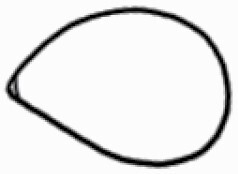	0.38 ± 0.38	no data
	48.	*glandular* hair with large globular head, single stalk cell and no neck cell	S	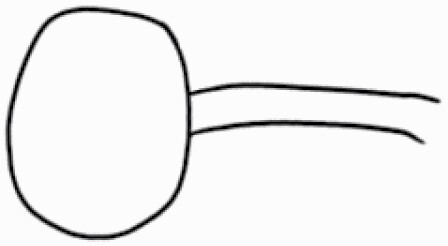	3.95 ± 1.03	118 ± 19.4 * 26.55 ± 1.82
	49.	porrect-stellate multiradiate *non-glandular* hair with subulate rays (4–5 in number) and with short central ray	C	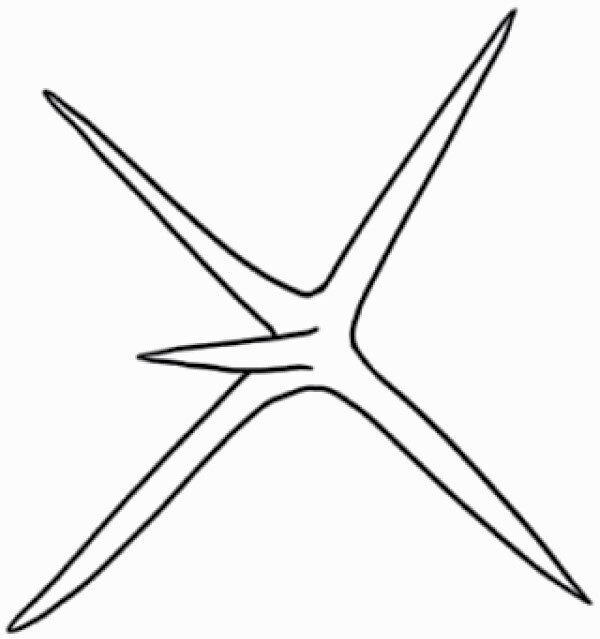	0.12 ± 0.12	127 ± 20.4
	50.	*non-glandular* hair with attenuate non-glandular branches and one gland-tipped branch	C	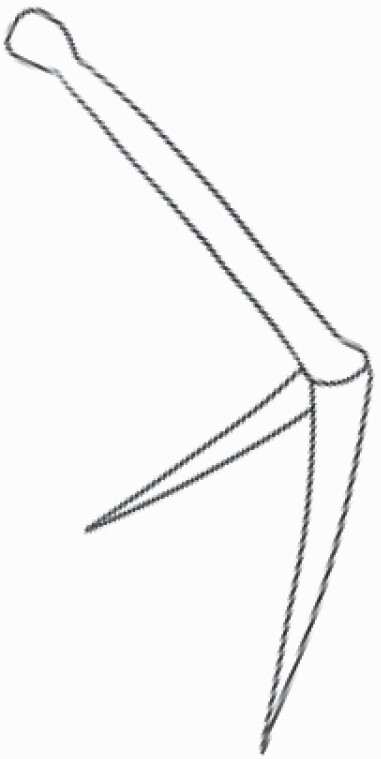	0.06 ± 0.06	201 * 39.4
Porcupine tomato (*Solanum pyracanthos*)	51.	porrect-stellate multiradiate *non-glandular* hair with subulate rays (2–12 in number) and with short central ray and pedestal	C	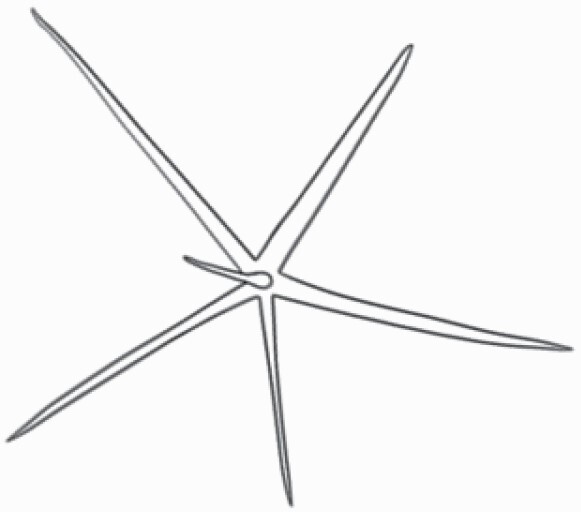	7.08 ± 1.50 (#51+ #53)	265 ± 8.5 (#51 and #53)
	52.	*glandular* hair with large globular head	S	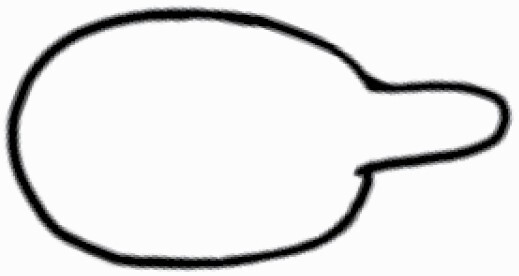	0.53 ± 0.24	55 ± 5.2 * 30.1 ± 3.29
	53.	bifurcated *non-glandular* hair with subulate rays with pulvinate multicellular base	C	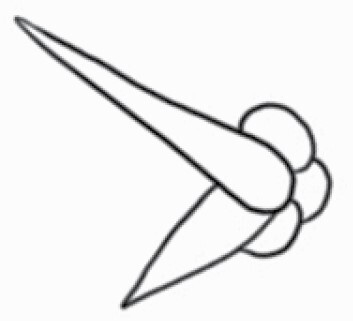	see #51	see #51
	54.	hooked *non-glandular* hair	S	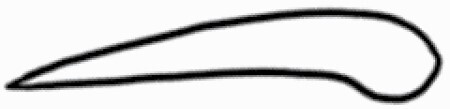	0.25 ± 0.14	126 ± 6.5
Bittersweet nightshade (*Solanum dulcamara*)	55.	*glandular* hair with small globular head	S	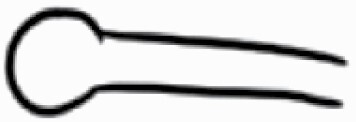	3.2 ± 0.33	86.7 ± 5.25 * 28 ± 1.65
	56.	attenuate basilatus *glandular* hair with small glandular tip	S	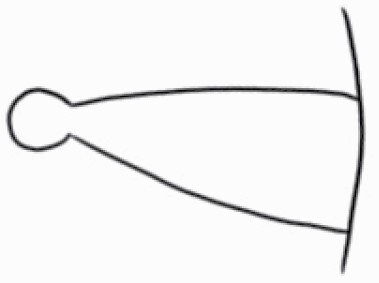	0.04 ± 0.04	no data
	57.	osteolate *non-glandular* hair with multicellular stalk	P	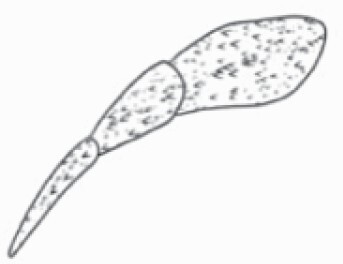	0.07 ± 0.05	no data

## Results

### Trichome morphology assessment

Basic classification of trichomes includes classifying them into glandular and non-glandular types (italicized; Tables 2 and 3). Here, detailed classification of trichomes was carried out using previously published works as a composite reference. The terminology used for nomenclature of trichomes is described below in Table 1.

Using above-mentioned terminology, we classified all the trichomes found in our samples. Although, three trichome types viz. stellate non-glandular, simple non-glandular and glandular type trichomes were the most found in the *Solanum* species in our study, these types have also been further characterized into a numerous subtypes based on minor morphological differences (Fig. 1; Tables 2 and 3).

Stellate non-glandular trichomes have further been divided into multitangulate, multiradiate, stellate non-glandular hair with subulate rays (2–6 in number) with the presence of pedestal (*Solanum aethiopicum*; adaxial) (Table 2; Serial number 2), porrect-stellate multiradiate non-glandular hair with subulate rays (varying in number) and with short central ray (*S. aethiopicum*; abaxial, *S. anguivi*; abaxial, *S. lanceifolium*; adaxial and abaxial, *S. ovigerum*; abaxial, *S. pyracanthos*; adaxial and abaxial) (Table 2; 9, 45; Table 3; 4, 7, 13, 45, 51), porrect-stellate multiradiate non-glandular hair with subulate rays (varying in number) with long central ray (*S. anguivi*; adaxial and abaxial, *S. lanceifolium*; adaxial, *S. ovigerum*; adaxial) (Table 2; 8, 10, 39; Table 3; 8), porrect-stellate multiradiate cruciate non-glandular hair with subulate rays (4 in number) and with short central ray (*S. grandiflorum*; abaxial) (Table 3; 30), porrect-geminate stellate multiradiate non-glandular hair with subulate rays (2–16 in number) and short central ray (*S. melongena*; adaxial and abaxial) (Table 2; 30; Table 3; 34). Moreover, some stellate trichomes also have a pedestal (*S. aethipicum*; adaxial, *S. anguivi*; adaxial and abaxial, *S. ovigerum*; abaxial, *S. pyracanthos*; abaxial) (Table 2; 2, 3, 8; Table 3; 7, 8, 45, 51). And some stellate trichomes with only two rays at an angle have been named separately as bifurcated non-glandular hair (*S. aethiopicum*; adaxial, *S. anguivi*; adaxial and abaxial, *S. lanceifolium*; abaxial, *S. pyracanthos*; abaxial) (Table 2; 3, 7; Table 3; 9, 14, 53). Within each stellate trichome, spike number also varied in almost all the species (Fig. 1; Tables 2 and 3).

Simple non-glandular trichomes have also been further subdivided as osteolate (*S. dulcamara*; abaxial) (Table 3; 57), subulate (*S. aethiopicum*; adaxial and abaxial, *S. anguivi*; adaxial and abaxial, *S. lycopersicum*; abaxial, *S. macrocarpon*; adaxial and abaxial, *S. melanocerasum*; adaxial, *S. grandiflorum*; adaxial and abaxial, *S. melongena*; adaxial and abaxial, *S. taeniotrichum*; abaxial, *S. caripense*; adaxial and abaxial, *S. ovigerum*; adaxial, *S. pyracathos*; adaxial, *S. dulcamara*; adaxial) (Table 2; 1, 6, 20, 22, 25, 31, 36, 37, 40; Table 3; 3, 6, 18, 23, 27, 33, 39, 40, 43), falcate (*S. grandiflorum*; abaxial, *S. ovigerum*; abaxial) (Table 3; 32, 44), setiform (*S. grandiflorum*; adaxial) (Table 2; 29), crescent (*S. melanocerasum*; abaxial) (Table 3; 24), attenuate (*S. lycopersicum*; adaxial and abaxial) (Table 2; 17, 18; Table 3; 19, 20), hooked (*S. aethiopicum*; abaxial; *S. lycopersicum*; adaxial and abaxial, *S. melongena*; abaxial, *S. caripense*; abaxial; *S. pyracanthos*; abaxial) (Table 2; 3, 16; Table 3; 18, 33, 40, 54). And most of these trichome types were smooth (all species) (Figs 1 and 2; Tables 2 and 3) while few others were pustulated (*S. macrocarpon*; adaxial and abaxial, *S. dulcamara*; abaxial) (Table 2; 20; Table 3; 23, 57). Similar to stellate non-glandular trichomes, some simple non-glandular trichomes have pedestal (*S. macrocarpon*; adaxial and abaxial, *S. grandiflorum*; adaxial, *S. melongena*; adaxial, *S. asperolanatum*; adaxial (Table 2; 20, 25, 29, 31, 41; Table 3; 23, 27), while others do not have pedestal.

The third most found trichome type was glandular type which has been further characterized based on the presence of a globular head (all species) (Figs 1 and 2; Tables 2 and 3) or small glandular tip (*S. aethiopicum*; abaxial, *S. lanceifolium*; abaxial, *S. lycopersicum*; adaxial and abaxial, *S. macrocarpon*; adaxial, *S. melanocerasum*; adaxial and abaxial, *S. grandiflorum*; adaxial, *S. taeniotrichum*; adaxial and abaxial, *S. asperolanatum*; adaxial and abaxial, *S. dulcamara*; abaxial) (Figs 2 and 3; Table 2; 13, 19, 23, 28, 33; Table 3; 1, 10, 17, 20, 25, 36, 37, 56). Further globular head can be large (Table 2; 5, 11, 12, 14, 21, 24, 26, 32, 41; Table 3; 2, 5, 12, 15, 16, 22, 26, 28, 31, 35, 42, 48, 52) or small (Table 2; 4, 5, 34, 35, 38, 44, 48; Table 3; 38, 46, 55), the characterization made based on comparative visual observations (Figs 2 and 3; Tables 2 and 3). Globular-headed trichomes can vary in shape as was observed in case of *S. lanceifolium* (doliform globular head; adaxial; Table 2; 9). Additionally, globular-headed glandular trichomes having distinct four-celled head have also been observed (Table 2; 5; Table 3; 5, 15, 16, 42). Similar to simple non-glandular trichomes, glandular trichomes were also found in various major shapes such as attenuate (*S. aethiopicum*; abaxial, *S. lanciefolium*; abaxial, *S. lycopersicum*; adaxial and abaxial, *S. macrocarpon*; adaxial, *S. grandiflorum*; adaxial, *S. dulcamara*; abaxial) (Table 2; 13, 19, 28; Table 3; 1, 10, 20, 57), acuminate (*S. lycopersicum*; adaxial) (Table 2; 15), subulate (*S. lycopersicum*; abaxial, *S. melanocerasum*; adaxial and abaxial, *S. taeniotrichum*; adaxial and abaxial) (Table 2; 23, 33; Table 3; 17, 25, 36, 37), hooked (*S. lycopersicum*; abaxial) (Table 3; 17). In case of non-glandular hair with attenuate non-glandular branches and one gland-tipped branch in *S. asperolanatum*, trichome has one branch with a glandular tip making it both glandular and non-glandular type trichome but named as non-glandular because of a greater number of rays being non-glandular (Table 3; 50).

And based on previous literature (Werker 2000) and since these trichome lack a clear distinction of glandular head, ovoid (*S. grandiflorum*; adaxial and adaxial, *S. asperolanatum*; adaxial and abaxial) (Fig. 1; Table 2; 27, 42; Table 3; 29), mamilla (*S. lycopersicum*; abaxial) (Table 3; 21) and verrucate (*S. lanceifolium*; abaxial) (Table 3; 11) trichomes were characterized as non-glandular. Although DSEM used in the study had numerous benefits including no sample preparation or the use of critical chemicals and machinery, and faster image processing (critical point dryers and sputter coaters; Kariyat *et al.* 2017), it also had some drawbacks. One of them was that we were unable to further classify the glandular trichomes based on the number of their head cells as SEM images lacked those details. And, since fresh leaf samples were used for the study, few glandular trichomes with pliable heads burst on their encounter with vacuum of the machine while scanning, due to the lack of critical point drying and sputter coating. For detailed results of species, trichome type, their density and dimensions with pictorial representation of each trichome type on adaxial leaf surface, see Table 2, and on abaxial leaf surface, see Table 3.

### Density measurements

Consistent with morphological diversity and variation among and within each species, density of trichomes also varied across species (Watts and Kariyat 2021; Figs 2 and 3; Tables 2 and 3). Oddly, in some of the species, while acquiring images for density count at 60×, we did not observe any trichomes, but while zooming in on different leaf samples at a higher magnification, we observed few trichome types although they were quite rare. These include attenuate basilatus glandular hair with small glandular tip on adaxial leaf surface of *S. macrocarpon* (Table 2; Serial number 19), attenuate basilatus glandular hair with small glandular tip; mamilla non-glandular hair on abaxial leaf surface of *S. lycopersicum* (Table 3; 20, 21), subulate *glandular* hair with multicellular jointed stalk and small glandular tip on abaxial leaf surface of *S. melanocerasum* (Table 3; 24), subulate *non-glandular* hair with multiseriate base and tall pedestal on abaxial leaf surface of *S. grandiflorum* (Table 3; 27), hooked subulate *non-glandular* hair on abaxial leaf surface of *S. caripense* (Table 3; 40) and falcate *non-glandular* hair on abaxial leaf surface of *S. ovigerum* (Table 3; 44).

The trichome types with highest trichome density include glandular hair with large globular head, single stalk cell and no neck cell on adaxial leaf surface of *S. asperolanatum* (Fig. 3; Table 2; 41), and porrect-stellate multiradiate *non-glandular* hair with subulate rays (5–10 in number) and with short central ray on abaxial leaf surface of *S. aethiopicum* (Fig. 2; Table 3; 4). Although the variation was huge, we found that some trichome types had higher trichome density than the other types in each species. For example, in *S. anguivi* (abaxial) porrect-stellate multiradiate non-glandular hair with subulate rays (5–10 in number) with short/long central ray and pedestal (density: 13.00 ± 1.56; Table 3; 7, 8 and 9) had considerably higher trichome density than the other two trichome types (Table 3; 5 and 6) (Fig. 2). This pattern was observed in almost all the species in which one/two trichome types dominated over the other. In addition to this, occasionally it was difficult at 60× to distinguish between some trichome types, so the density of some trichome types has been compiled. For example, manual counting of both glandular hair with large quadricellular globular head and single stalk cell in case of abaxial leaf surface of *S. lycopersicum* resulted into total density of 0.94 ± 0.19 (Table 3; 15 and 16). Such cases were observed for all three major trichome types (stellate non-glandular, simple non-glandular and glandular trichomes) (Tables 2 and 3). Additionally, we found significant variation in trichome numbers (total, glandular and non-glandular; at 60× magnification) among species (generalized regression; *P* ≤ 0.0001) and interaction of species with trichome type (total, glandular and non-glandular) (generalized regression; *P* ≤ 0.0001) (Figs 4–6), but the variation was non-significant between trichome types (generalized regression; *P* = 0.6971).

### Trichome dimensions

Similar to density, dimensions of each trichome type also varied across species and location (Fig. 7; Tables 2 and 3). Among non-glandular trichome types, subulate non-glandular hair with multiseriate base and tall pedestal on abaxial leaf surface of *S. grandiflorum* (Table 3; Serial number 27) was the longest in dimensions, and the shortest non-glandular trichome was subulate non-glandular hair with multicellular jointed stalk and multicellular base on abaxial leaf surface of *S. ovigerum* (Table 3; 43). Longest glandular trichome type was subulate glandular hair with multicellular jointed stalk, multicellular base, distinct subsidiary cells and small glandular tip on adaxial leaf surface of *S. melanocerasum* (Table 2; 23), and the shortest glandular trichome type was glandular hair with large globular head on abaxial leaf surface of *S. melongena* (Table 3; 35). The glandular trichome with largest head was glandular hair with large quadricellular globular head and single stalk cell on abaxial leaf surface of *S. lycopersicum* (Table 3; 16). The glandular trichome with smallest glandular tip was hooked subulate glandular hair with multicellular jointed stalk and small glandular tip on abaxial leaf surface of *S. lycopersicum* (Table 3; 17). Dimensions of some trichome types seemed comparable and thus dimension data for few trichome types were collected as one type (stellate and bifid trichomes; all globular glandular trichomes; glandular trichomes with small tip; simple trichomes).

Although the stellate trichomes had almost consistent spike length within a species or/and leaf surface, but central ray of stellate trichomes varied (long/short) resulting into subdividing them into stellate trichomes with short or long central ray. Simple trichomes had the most variation resulting them being both the shortest and the longest trichome found among species in the study. Further, in general, glandular trichomes with globular heads were shorter than glandular trichomes with small tip on the top. Additionally, glandular trichomes with globular heads had greater diameter of their glandular heads than glandular trichomes with small tip on the top. Due to the high species numbers, processing of multiple samples and incredible trichome diversity, we could not acquire dimensions of some trichome types (e.g. osteolate non-glandular hair with multicellular stalk on abaxial leaf surface of *S. dulcamara*; Table 3; 57).

## Discussion

In this study, we examined the trichome characteristics including their nomenclature, density and dimensions on both adaxial and abaxial leaf surface of 14 *Solanum* species using scanning electron microscopy on fresh leaf samples. We found that *Solanum* genus consists of numerous trichome types which vary not only among the species, but also within each species and between adaxial and abaxial leaf surfaces. We also found that three trichome types are most common in *Solanum*: stellate non-glandular, simple non-glandular and glandular trichomes. Broadly, all trichomes have been characterized into glandular and non-glandular trichomes, but it is not fair, because both glandular and non-glandular trichomes can further be classified into various types based on their shape, size, number of cells, basal cells, neck cells, etc. Besides, it was not certain whether ovoid, verrucate and mamilla trichomes are glandular or non-glandular because of their unusual and perplexing structure but we confirm that they can be characterized as non-glandular trichomes because of their lack of distinction of a clear glandular head.

Trichomes, in general, have been proven to be an excellent phenotypic trait for finding evolutionary and taxonomic relationships among species (Cantino 1990; Eyvazadeh Khosroshahi and Salmaki 2019). For example, *Phlomis* genus has characteristic multi-nodal-branched trichomes and can be considered as synapomorphy for this group, but *Phlomoides* lacks this feature and thus, this feature most likely represents a plesiomorphy in the genus (Eyvazadeh Khosroshahi and Salmaki 2019); and separation of African and Asian *Leucas* spp. was made more explicit by the differences of capitate trichomes with definite morphology and absence of non-glandular trichomes with one cell and more than three cells (Mannethody and Purayidathkandy 2018). Our study characterized the finer details of trichome morphology, and it can be of an aid in exploring phylogenetic and taxonomic relationships among the members of genus *Solanum*, and their relationship with members of other genus of Solanaceae family and among other plant families. Additionally, trichomes act as excellent cell differentiation models (Hülskamp 2004) and provided with the diversity from this study, it can be explored how differentiation in trichome cells results into production of glandular/non-glandular of various shapes and sizes.

Defence against herbivores is one of the major functions of trichomes, and both glandular and non-glandular trichomes have been well documented to deter herbivore movement and feeding (Kariyat *et al.* 2013, 2017, 2019). Morphology, density and dimensions relationships of subtypes of trichomes can be employed to find correlations between trichome characteristics with herbivore feeding intensity and behaviour (Li *et al.* 2020). For instance, Watts and Kariyat (2021) found that significantly higher trichome density on abaxial leaf surface than adaxial leaf surface of Solanaceae species resulted into delayed feeding and lower mass gain of tobacco hornworm caterpillars (*Manduca sexta*; Lepidoptera: Sphingidae); Kariyat *et al.* (2017) found damage done to peritrophic membrane (gut lining) of *M. sexta* caterpillars after feeding on stellate trichomes of horsenettle (*S. carolinense*; Solanaceae). Further, since the damage done by non-glandular trichomes is primarily because of their structure and many herbivores mow the trichomes off the plant surface before feeding (Kariyat *et al.* 2017, 2019; Kaur and Kariyat 2020b), we speculate that trichomes with a greater number of spikes, and spikes with higher dimensions can result into higher negative impacts on herbivore feeding, but warrant closer examination (Medeiros and Moreira 2005; Andama *et al.* 2020). The variation in trichome types, density, dimensions and their functional consequences (Watts and Kariyat 2021) between abaxial and adaxial leaf surfaces also warrants detailed exploration, a reason why we examined these differences in detail (Tables 2 and 3). Contrary to non-glandular trichomes, glandular trichomes are the secretory structures and contain various types of chemicals in their head cells and those chemicals have been found to trigger different defence-related pathways against herbivores (Tissier 2012), and some glandular trichome types have been found to play more prominent roles than the others. For example, type VI (as named by Luckwill 1943; glandular trichomes with quadricellular head) trichomes of *Lycopersicon* genus were found to contain chemicals possessing insecticidal properties against lepidopteran larvae (Lin *et al.* 1987). Thus, knowledge of glandular trichome density and dimensions such as length and diameter can help us know the plant parts with higher trichome density, amount of chemicals possessed by trichomes and if the trichomes are tall enough to act against herbivores with its structural features along with chemical defence. Additionally, expanding on this study, histochemistry and volatile collection of various glandular secretions can be done, and anti-herbivore chemicals can be identified (Muravnik *et al.* 2019), and can lead to further functional assessment and classification, an area that we are currently exploring. Trichomes have been of great importance against herbivores as a defence trait, and thus, have been incorporated in integrated pest management of insect pests of various crops of economic importance including potato (*Solanum tuberosum*; Solanaceae), cotton (*Gossypium* spp.; Malvaceae), cowpea (*Vigna unguiculata*; Fabaceae), to name a few. For instance, *hooked* non-glandular trichomes of *Phaseolus vulgaris* (Fabaceae) entrap pests such as black bean aphid (*Aphis fabae*; Hemiptera: Aphididae) and green stink bug (*Nezara viridula*; Hemiptera: Pentatomidae) (Rebora *et al.* 2020). Trichomes also play multiple roles in plants including leaf water uptake and protection from UV light, in addition to defence against herbivores (Sack and Buckley 2020). For example, Li *et al.* (2021) showed that among three trichome types viz. non-glandular trichomes, linear glandular trichomes and glandular trichomes, only non-glandular trichomes of Sunflower (*Helianthus annuus*) were found to accumulate and translocate zinc, an important micronutrient for plants, a potential area of research to be explored for mineral and nutrient uptake to enhance crop yield through trichomes. Clearly, trichome characteristics have the potential to be explored in relation to plant ecophysiological functions such as water use efficiency (Thitz *et al.* 2017), UV protection and mineral uptake.

Taken together, this study documented the variation in trichome types, their density and dimensions in representative *Solanum* species. So far, the major reasons which withheld the detailed classification and nomenclature of trichomes were (i) the conventional approach to classify trichomes as just glandular or non-glandular trichomes failed to document their morphotypes and function, (ii) the requirement of expensive machinery and skilled professionals, required to process samples across species and families and (iii) the need of intensive and detailed workflow for the closer examination of images to extract all the data on morphology, density and dimensions. By overcoming these challenges, we show the variation in trichome traits within a subset of the genus *Solanum* and encourage more detailed examination across various plant families.

## Conclusions

Overall, the study provides unusually fine details and morphological characterization of trichomes of a mixture of wild and domesticated, annual, and perennial, food crops and weed species of genus *Solanum* which can act as referral source for further studies of most of trichome-related parameters and their relationships with biotic and abiotic stresses (Tian 2012). Since *Solanum* is the largest genus of one of the major angiosperm family viz. Solanaceae, exploring trichome diversity by considering 14 species from various groups (e.g. *S. macrocarpon* and *S. lycopersicum* are cultivated species, while *S. anguivi* and *S. pyracanthos* are wild species) of the family provided us with an updated data source of trichome characteristics with such details that never has been done before. Further, future directions in trichome studies can be focused on understanding variability and organ development while studying gene expression simultaneously using trichomes as a model. Moreover, trichomes are also known to play role in multi-trophic interactions in ecosystem (Weinhold and Baldwin 2011) and thus, each trichome type can be explored for its potential in strengthening plants’ defences.

## Notes

**Form & Function. Chief Editor:** Kate McCulloh
